# Mechanistic modeling with a variational autoencoder for multimodal single-cell RNA sequencing data

**DOI:** 10.1101/2023.01.13.523995

**Published:** 2023-01-14

**Authors:** Maria Carilli, Gennady Gorin, Yongin Choi, Tara Chari, Lior Pachter

**Affiliations:** 1 Division of Biology and Biological Engineering, California Institute of Technology; 2 Division of Chemistry and Chemical Engineering, California Institute of Technology; 3 Biomedical Engineering Graduate Group, University of California, Davis; 4 Genome Center, University of California, Davis; 5 Department of Computing and Mathematical Sciences, California Institute of Technology

## Abstract

We motivate and present *biVI*, which combines the variational autoencoder framework of *scVI* with biophysically motivated, bivariate models for nascent and mature RNA distributions. In simulated benchmarking, *biVI* accurately recapitulates key properties of interest, including cell type structure, parameter values, and copy number distributions. In biological datasets, *biVI* provides a route for the identification of the biophysical mechanisms underlying differential expression. The analytical approach outlines a generalizable strategy for representing multimodal datasets generated by single-cell RNA sequencing.

## 1 Introduction

Advances in experimental methods for single-cell RNA sequencing (scRNA-seq) allow for the simultaneous quantification of multiple cellular species at a time, such as nascent and mature transcriptomes [1, 2], surface [3-5] and nuclear [6] proteomes, and chromatin accessibility [7, 8]. The biophysical “integration” of such datasets requires the parameterization of interpretable mechanistic models [9]. Such modeling is challenging because mechanistic models tend to be intractable for more than a few co-regulated genes; in practice, single-cell genomics measurements are made for thousands of genes in tens of thousands of cells [10].

One approach to integration of multimodal single-cell genomics data has been to leverage recent advances in machine learning [11-13] and matrix factorization techniques [14]. This approach aims to summarize multimodal single cell measurements in a common low-dimensional space, which purports to reflect the cell state structure. Specifically, single-cell variational inference (*scVI*), a popular machine learning framework for analyzing scRNA-seq data [12, 15], has previously been adapted to multimodal data with protein [11] and chromatin [16] measurements. However, methods based on this approach are neither informed by nor lead to specific biophysical interpretations, and omit the intrinsic causal relationships between “upstream” and “downstream” parts of the central dogma [17, 18].

**Figure 1:**
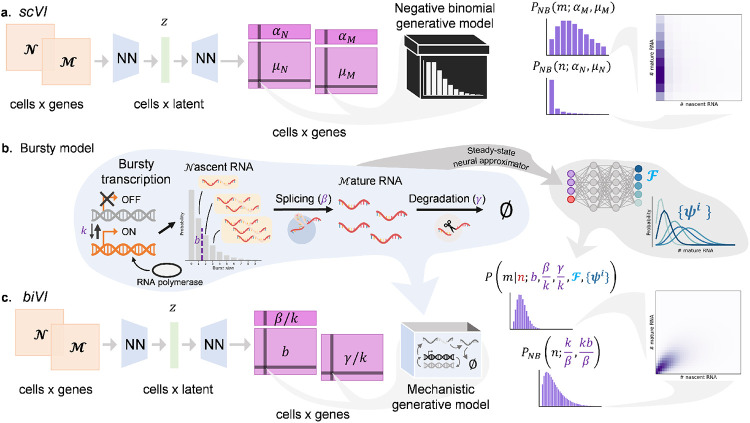
*biVI* reinterprets and extends *scVI* to infer biophysical parameters rather than statistical summaries. **a.** *scVI* can take in concatenated nascent (𝒩) and mature (ℳ) RNA count matrices, encode each cell with a common low-dimensional representation *z*, and learn the per-cell parameters *μ_N_* and *μ_M_* and per-gene parameters *α_N_* and *α_M_* that encode formally independent nascent and mature count distributions. This approach is descriptive and not motivated by any specific model of physiology. **b.** A schematic of the telegraph model of transcription: a gene locus has the on rate *k*, the off rate *k_off_*, and the RNA polymerase binding rate *k*_RNAP_. In the bursty regime, where *k*_RNAP_ and *k_off_* are relatively high, nascent RNA molecules are produced in geometrically distributed bursts with mean *b* = *k*_RNAP_/*k_off_*. After production, molecules are spliced at a constant rate *β* and degraded at a constant rate *γ*. Although the two-stage telegraph model does not have a closed-form solution, its steady-state distribution can be approximated with the combination of a pre-trained neural network ℱ and a set of basis functions {*ψ*^*i*^}. **c.** *biVI* can take in nascent and mature count matrices, produce a common low-dimensional representation for each cell, and output per-cell parameters *b* and *γ/k*, as well as the per-gene parameters *β/k*, for a mechanistically motivated joint distribution of nascent and mature counts.

Consider, for example, the joint modeling of nascent and mature RNA counts, which are readily available by realigning existing scRNA-seq reads [1, 2], via the *scVI* framework. While *scVI* has not been considered for such integration, one can conceive of utilizing it for this purpose as follows: each cell could be encoded by a neural network to a latent low-dimensional vector *z*, which could be decoded into a set of cell- and gene-specific parameters by another neural network. These parameters would define a negative binomial distribution used to evaluate and optimize the likelihoods of RNA count matrices. In principle, nascent and mature RNA molecules can be “integrated” with *scVI* by concatenating the two matrices and treating nascent and mature transcripts as distinct “genes.” Latent representations of cells would then be decoded to produce parameters for independent distributions of nascent and mature molecules, as diagrammed in Figure 1a. However, the likelihoods and inferred parameters would have no biophysical interpretation and would merely be represented as part of a neural “black box” used to reduce data dimensionality.

Here, we introduce *biVI*, a strategy that adapts the variational autoencoder (VAE) framework to work with well-characterized stochastic models of transcription, and use it to fit simulated and biological datasets that include nascent and mature RNA counts. First, we propose several stochastic models of transcription, formalized by chemical master equations (CMEs), that could give rise to count distributions consistent with *scVI*. For example, Figure 1b illustrates the bursty model of transcription, in which nascent RNA molecules are produced in geometrically distributed bursts with mean *b*, which arrive at a constant rate *k*. Nascent molecules undergo splicing at rate *β* to produce mature molecules; these are, in turn, degraded with constant rate γ. The bursty model has been extensively experimentally validated in mammalian cells [19-21], and gives rise to a joint distribution of nascent and mature molecules parameterized by the burst size and the relative rates of transcription, splicing, and degradation. While the joint steady-state distribution induced by the bursty model is analytically intractable [22], we have previously shown that it can be efficiently and accurately approximated by the combination of judiciously chosen basis functions and a pre-trained neural network designed to predict their weights [23]. *biVI* replaces *scVI*’s independent nascent and mature likelihoods with mechanistically motivated, *b*iologically *i*nterpretable *bi*variate likelihoods. The method encodes a concatenated matrix of nascent and mature counts to a low-dimensional representation, which in turn decodes to parameters which have biophysically meaningful interpretations under a particular model (Figure 1c). Using *biVI*, we can thus infer biophysical parameters for mechanistic models of transcription for tens of thousands of single cells and genes. While we focus our analysis on the bursty model, *biVI* implements two alternative descriptions of single-molecule variation, the constitutive and extrinsic noise models previously discussed in the literature [9,24,25]. The descriptions and derivations of each biophysical model are available in Section S1, with diagrams for the constitutive model in Figure S1 and extrinsic model in Figure S2.

## 2 Results and discussion

Before using *biVI* to infer biophysical parameters with experimental data, we sought to evaluate its ability to reconstruct distributions from a known mechanistic ground truth. By showing that *biVI* can accurately recapitulate simulated data distributions, we can increase confidence in the validity of biophysical parameters inferred from experimental data. The simulation procedure is outlined in Section 4.5, with further details in Section S2. For the bursty model, we generated parameters *b*, *β/k*, and γ/*k* for 2,000 genes, increasing burst sizes for marker genes to produce twenty distinct cell types (Section S2). Next, we sampled nascent and mature RNA counts for 10,000 cells, approximately equally distributed across cell types. We trained *biVI* with the bivariate bursty likelihood and *scVI* with independent negative binomial likelihoods on 80% of the simulated cells (Section 4.5), holding out 20% for testing. To reconstruct distributions for a given gene in a specific cell type, we took the average of the probability laws obtained for that cell type (Section 4.5.3). We reproduced the analysis for the constitutive and extrinsic models, with results shown in Figures S4 and S5.

**Figure 2:**
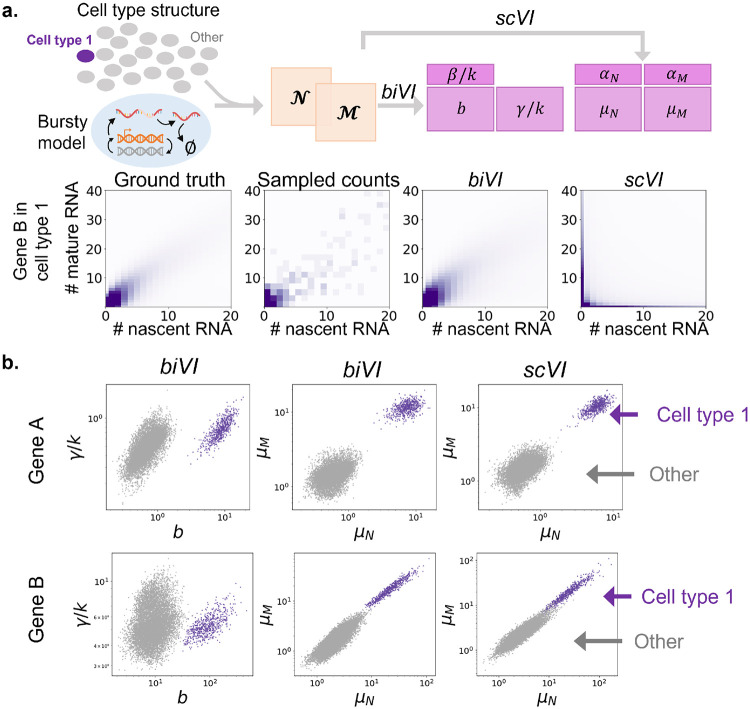
*biVI* reflects the global structure and stochasticity of simulated bursty data. **a.** We generated parameters for 2,000 genes under the bursty model of transcription, and sampled nascent and mature counts from the resulting joint distribution to yield 10,000 cells across 20 cell types. Next, we trained *biVI* with the bivariate bursty likelihood and *scVI* with independent negative binomial likelihoods. For a sample gene, *biVI* accurately reconstructs the underlying bivariate distribution (darker color: higher; lighter color: lower probability mass or number of observations). **b.** Cell-specific parameters inferred by *biVI* and *scVI* for two marker genes for cell type 1, Gene A and Gene B. *biVI* yields nascent and mature means, as well as parameter values, which are related to the means through a transformation. *scVI* only yields the means.

On simulated data generated with the bursty model, the negative log-likelihood, or reconstruction loss, of the 2,000 held-out testing cells was lower for *biVI* (3,885.8) than for *scVI* (3,953.6) (Figure S3). Applying *biVI* to fit the model used to generate the data set gave lower reconstruction losses than *scVI* for all three simulated data sets (Figure S4, Figure S5). Furthermore, the Kullback-Liebler divergence (KLD) between true and reconstructed count distributions, separately computed for each cell type, was lower for *biVI* (0.014 on average) than *scVI* (0.212 on average) (Figure S3). Figure 2a qualitatively illustrates *biVI*’s improved ability to reconstruct distributions based on noisy sampled observations. If the model is correct, *biVI* achieves better reconstruction performance on several metrics. Conversely, the suboptimal results under the *incorrect* models suggest that *biVI* may provide a route for model selection.

A primary use of *scVI* is to reduce the dimensionality of similar cells, such as multiple observations of the same cell type, to similar low-dimensional latent representations, removing noise and redundancies. *biVI* facilitates this application for multimodal data. To characterize how well cell types are preserved in the latent space, we applied several clustering metrics (Section S4) to the latent representation of 2,000 cells (simulated, bursty model, held-out set) encoded by *biVI* and *scVI* (Section S2), using the ground truth cell types as cluster assignments. The average percent of latent-space nearest neighbors in the same cell type as each cell, computed over all cells, was similar for *biVI* (90.0%) and *scVI* (90.4%). The silhouette score, a measure of how similar each cell is to other cells in its cluster compared to other clusters, achieved analogous performance, yielding 0.383 for *biVI* and 0.387 for *scVI*. Finally, scaled inter-cluster distance (average inter-cluster distance divided by average intra-cluster distance, with larger values corresponding to better cluster separation), was 2.75 for *biVI* and 2.60 for *scVI*. These clustering metrics are evidence that cells can be similarly well represented in a low dimensional space using *biVI* and *scVI*, and that *biVI* modifications do not impede the process of variational inference or downstream analyses.

By incorporating nascent data, *biVI* enables the detection of interesting differences in gene expression that may be subtle or poorly identifiable from mature RNA counts alone. By using a specific biophysical model, it provides an explanatory framework. Figure 2b shows inferred parameters across all simulated cells for two marker genes, which have higher burst sizes in the simulated cell type 1. The first column plots the *biVI* inferred burst size *b* against relative degradation rate γ/*k* for every cell, the second column displays *biVI* inferred mature means *μ_M_* and nascent means *μ_N_*, whereas the third displays the *scVI* inferred means. The *biVI* means and parameters are related through the transformations outlined in Section 4.9. Gene A shows a clear separation of cell type 1 in all workflows. In contrast, Gene B shows a clearer separation of parameter values than averages, and correctly ascribes it to a change in burst size.

With these simulation benchmarks suggesting that *biVI* can perform as well as or better than *scVI*, we proceeded to use the two pipelines to analyze experimental data. We selected a single scRNA-seq library generated from mouse brain tissue [26] and processed it with *kallisto*∣*bustools* [2] to obtain spliced and unspliced count matrices (Section 4.7). These count matrices contained the data for 6,418 previously annotated cells across 19 cell subtypes with expression measurements for the 2,000 most highly variable genes (Section 4.7). Next, we fit the counts with *biVI* (Section 4.8), making the key assumption that unspliced and spliced molecules may be treated as the nascent and mature species of the bursty generative model. 4,622 cells were used to train both VAE models with 513 validation cells, with 1,283 testing cells held out for assessing performance.

**Figure 3:**
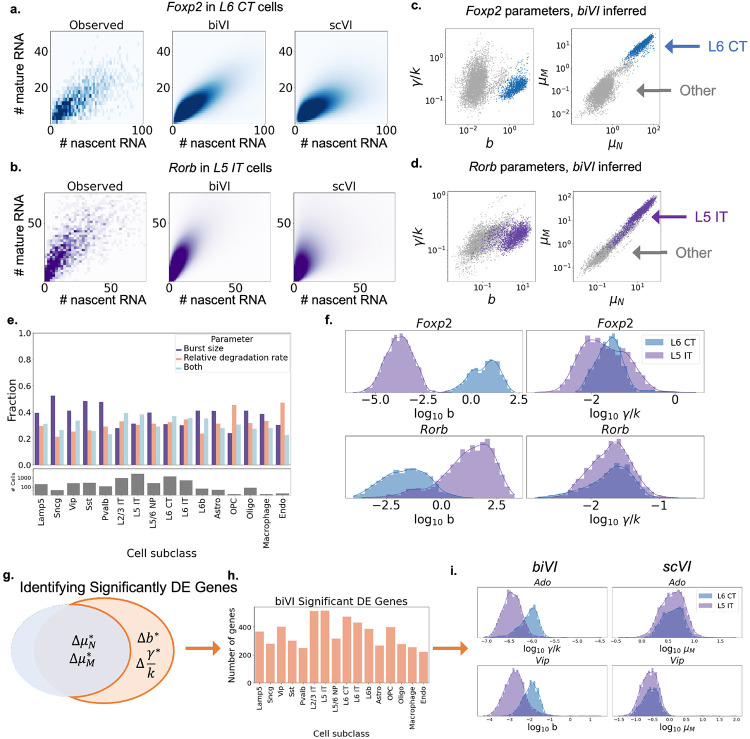
*biVI* successfully fits single-cell neuron data and suggests the biophysical basis for expression differences. **a.-b.** Observed and inferred distributions of *Foxp2*, a marker gene for L6 CT cells, and Rorb, a marker gene for L5 IT cells (conventions as in Figure 2a). **c.-d.** Cell-specific parameters inferred for *Foxp2* and *Rorb* demonstrate identifiable differences in means and parameters in the marked cell types. **e.** Cell subclasses show different modulation patterns, with especially pronounced distinctions in non-neuronal cells (top: fractions of genes exhibiting differences in each parameter; bottom: number of cells in each subclass). **f.** Inferred parameters for *Foxp2* and *Rorb* in L5 IT (purple) and L6 CT (blue) cells show consistent differences in burst size. **g.** *biVI* allows the identification of cells which exhibit differences in burst size or relative degradation rate, without necessarily demonstrating differences in mean expression. **h.** Hundreds of genes demonstrate this modulation behavior, albeit with variation across cell subclasses. **i.** Histograms of *biVI* parameters and *scVI* mature means for two genes that exhibit parameter modulation without identifiable mean modulation. *Ado* appears to show differences in the degradation rate, whereas *Vip* appears to show differences in the burst size (top: results for *Ado*; bottom: results for *Vip*; purple: L5 IT cells; blue: L6 CT cells).

Figure 3a-b shows the observed and reconstructed distributions of *Foxp2* (an L6 CT neuron marker), and *Rorb* (an L5 IT neuron marker), restricted to their respective cell types. Consistently with simulations, qualitative visual inspection suggests that *biVI* recapitulates distribution shapes better than *scVI*.

In addition to accurately capturing observed distributions, we can interpret the inferred parameters to determine *how* genes are regulated. Figure 3c-d shows the decoded averages and parameters for *Foxp2* and *Rorb* across all cells. The average nascent and mature counts are higher in the cell types they mark, highlighted in corresponding colors. However, additionally, the parametric *biVI* results suggest these genes are upregulated at the transcriptional level, by increasing the burst size rather than decreasing the degradation rate.

We can leverage these differences to compare gene regulation trends across numerous cell types. We identified genes with significant differences in the parameters of one cell type versus all others using a two-sided *t*-test (Section 4.9), using a Bonferroni-corrected *p*-value threshold of 0.05 and a log_2_ fold change threshold of 1. To stay consistent with *scVI* [15], and avoid the conflation of “systematic” and “sequencing depth”-like effects, we evaluated differences in the compositional, “normalized” versions of inferred burst sizes, nascent means, and mature means, independent of the *scVI* cell size factor. We note that Figure 3 demonstrates the “scaled,” or non-normalized versions of these quantities. The relative degradation rate is independent of sequencing depth, so differential expression analysis was performed directly on the inferred values. To avoid the potential pitfalls of small sample sizes, we excluded all cell types with fewer than 10 barcodes (L5 ET, L6 IT Car3, VLMC, SMC) from this analysis. Figure 3e shows the fraction of identified genes in each cell subclass that exhibited differences in burst size, relative degradation rate, or both. While cell subclasses modify expression of genes through a variety of regulatory strategies (modifying burst size, degradation rate, or both), some interesting patterns can be observed. For example, the GABAergic subclasses (first five categories) appeared to more consistently exhibit deviation in burst size than in degradation rate. These coarse-grained differences may suggest common regulatory behaviors are present in similar cell types, although more sophisticated statistical methods are necessary to draw rigorous conclusions about these trends.

Finally, *biVI* identified novel genes that were significantly differential between cell subclasses in burst size or relative degradation rate but not nascent or mature inferred means (Section 4.9, Figure 3g-h). For some cell types, there were several hundred such genes, potentially interesting targets for follow-up experimental investigation. For example, the gene *Ado* demonstrated statistically significant differences in the *biVI* inferred relative degradation rate, but not *scVI* inferred mature means, in the neuronal subclass L6 CT (Figure 3i, top row). *Ado* has been associated with gliablastoma metabolism in non-neuronal cells [27]; the observation that it shows significant differences in inferred relative degradation rates in a neuronal cell subclass could lead to a broader understanding of the role its noise behaviors play in the growth and progression of malignant gliomas. As another example, burst size of *Vip*, the gene that codes for the peptide hormone vasoactive intestinal peptide (VIP), is statistically significantly different in neuronal cells of subclass L5 IT (Figure 3i, bottom row). While VIP is known to act as a hormone receptor and marks a GABAergic neuronal subclass (Vip cells) [28], the regulation of its burst size in (glutamatergic) L5 IT cells suggests functions in neurons outside of the subclass it marks, a suggestive area for follow-up investigation. Such mechanistic description provides a framework for characterizing the connection between the gene’s role and the cell’s regulatory strategies beyond the mean expression [29, 30].

## 3 Conclusion

We have demonstrated that a mechanistically interpretable autoencoder can be defined in a self-consistent way for bivariate data. This autoencoder, implemented in the *biVI* framework, combines the functional and distributional assumptions of *scVI* with the mechanistic hypotheses of transcriptional biophysics. When applied to simulated data, it correctly recovers three properties of interest. (1) Recapitulation of the gene-specific distributions, as characterized quantitatively through the KLD and qualitatively through the distribution shape. (2) Maintenance of cell type structure, as quantified by a variety of clustering metrics. (3) Recovery of the physical parameters used to generate count matrices, including the biophysical basis of differences in marker genes. When applied to real sequencing data, it achieves similar performance, and attains a degree of interpretability: we can begin to attribute differences in expression to specific regulatory mechanisms.

This interpretability is both enabled and limited by the modeling decisions: the biological findings are only as meaningful as the model. For simplicity and consistency with *scVI*, we have made several key assumptions. Most crucially, we propose that the model has been correctly specified, and that only the burst sizes and degradation rates are modulated between different cell types or states. It is likely that the latter assumption can be made somewhat more physically realistic by proposing that regulation primarily occurs at the gene locus, i.e., allowing the burst size *b* and burst frequency *k* to vary while keeping the processing rates *β* and γ constant. At this time, the most appropriate way to mathematically formulate this model while maintaining compatibility with neural networks is not yet clear. On the other hand, controlling for misspecification of the generative model appears to be a much more challenging problem, whether in terms of establishing a goodness-of-fit criterion or in terms of performing model selection simultaneously with inference. Preliminary comparisons, wherein we use multiple models to fit simulated datasets, suggest that the correct model may be identifiable, but the appropriate statistical procedures are not yet clear. Relaxing model assumptions and implementing these extensions are the next natural steps for the interpretable summary of large datasets.

More fundamentally, we have assumed that a variational autoencoder framework is *a priori* appropriate for single-cell RNA sequencing datasets. However, this framework is incompatible with certain common axioms [31]. For example, *scVI* and *biVI* cannot represent truly discrete cell types, i.e., sets of cells that have an identical latent representation but may have distinct count distributions due to single-molecule stochasticity. In addition, steady-state probability distributions are intrinsically incompatible with *transient* phenomena, such as differentiation (as discussed in [31]) and cell cycling, limiting the scope of this approach to stationary systems. Finally, we have merely asserted that the *biVI* mean parameters can be represented in a neural, compositional form, and demonstrated that this formulation produces acceptably faithful data summaries; however, we have not taken the next step of justifying or critiquing this assertion. To improve the prediction of nascent and mature count matrices, we have imposed biophysical constraints on the species’ joint distributions. Although the generative model is interpretable, the neural component is still a computational heuristic. In a similar spirit, but the orthogonal direction, previous work in the summary of single-cell data has used *linear* decoders, which constrain the compositional gene abundance parameters to be linear transformations of the latent embedding vector [32]. We anticipate that broader improvements in interpretability will require considerable further investigation and formalization, potentially with analogous, hypothesis-driven constraints on the variational autoencoder structure.

In spite of the limitations imposed by these assumptions, the approach we present in this report provides a fairly general, principled toolbox for the treatment of multimodal datasets. As we discuss in the mathematical treatment, the analytical procedure involves instantiating a set of biophysical hypotheses, analyzing the resulting stochastic system to obtain its distribution, and using this distribution as a multivariate generative model. As single-cell technologies evolve to provide larger-scale and more precise measurements of biomolecules, we anticipate that this approach can be self-consistently extended to provide a more comprehensive picture of biophysical processes in living cells.

## 4 Methods

In order to extend the *scVI* method to work with multimodal molecule count data in a way that is coherent with biology as well as the autoencoder, we define bivariate likelihood functions that (i) encode a specific, precedented mechanistic model of transcriptional regulation and (ii) are admissible under the assumptions made in the standard *scVI* pipeline. On a high level, our method entails the following steps:

1. Choose one of the *scVI* univariate generative models (Section 4.2), including the functional form of its likelihood and any assumptions about its distributional parameters.
2. Identify a one-species chemical master equation (CME) that produces this distribution as its steady state, and translate assumptions about distributional parameters into assumptions about the biophysical quantities that parameterize the CME (Section 4.3). The one-species system and its assumptions will typically not be uniquely determined.
3. Identify a two-species CME and derive assumptions about parameter values consistent with the one-species system. There will typically be multiple ways to preserve the assumptions but only a single CME.
4. Modify the autoencoder architecture to output the variables that parameterize the CME solution under the foregoing assumptions, and use this solution as the generative model.

### 4.1 Statistical preliminaries

We use the standard parameterization of the Poisson distribution:

(1) $$P_{\text{Poiss}}(x;\mu )=\frac{\mu^{x}e^{-\mu }}{x!}.$$

We use the shape-mean parameterization of the univariate negative binomial distribution:

(2) $$P_{\text{NB}}(x;\alpha ,\mu )=\frac{\Gamma (\alpha +x)}{x!\Gamma (\alpha )}{\left(\frac{\alpha }{\alpha +\mu }\right)}^{\alpha }{\left(\frac{\mu }{\alpha +\mu }\right)}^{x}.$$

We use mean parameterization of the geometric distribution on $N_{0}$:

(3) $$P_{\text{Geo}}(x;b)={\left(\frac{b}{b+1}\right)}^{x}\left(\frac{1}{b+1}\right).$$

### 4.2 *scVI* models

A brief summary of the generative process of the standard, univariate *scVI* pipeline is useful to contextualize the options and constraints of the bivariate model. Each cell is represented by a low-dimensional vector *z*, which is formally a random variable. *scVI* uses the “decoder” neural network to generate the quantities *ρ_cg_*, which describe the compositional abundance of gene *g* in cell *c* as a function of *z*, such that $\sum_{g}\rho_{cg}=1$. Furthermore, it either fits or uses a plug-in estimate for ℓ*_c_*, a cell-specific “size factor,” such that the mean expression of a gene in a given cell is *μ_cg_* = *ρ_cg_*ℓ*_c_*.

The univariate workflow provides the options of three discrete generative models: Poisson with mean *μ_cg_*, negative binomial with mean *μ_cg_* and gene-specific dispersion parameter *α_g_*, and zero-inflated negative binomial, with an additional Bernoulli mixture parameter. We report the master equation models consistent with the first two generative laws below, and discuss a potential basis for and reservations about the zero-inflated model in Section S1.4

### 4.3 Master equation models

The one-species CMEs encode reaction schema of the following type:

(4) $$\emptyset \to 𝒳\overset{\gamma }{\to }\emptyset ,$$

where 𝒳 is a generic transcript species used to instantiate a univariate *scVI* generative model, γ is the transcript’s Markovian degradation rate, and the specific dynamics of the transcription process (first arrow) are deliberately left unspecified for now. Such systems induce univariate probability laws of the form *P*(*x*).

The two-species CMEs encode reaction schema of the following type:

(5) $$\emptyset \to 𝒩\overset{\beta }{\to }ℳ\overset{\gamma }{\to }\emptyset ,$$

where 𝒩 denotes a *n*ascent species, ℳ denotes a *m*ature species, and *β* denotes the nascent species’ Markovian conversion rate. Such systems induce bivariate probability laws of the form *P*(*n,m*). We typically identify the nascent species with unspliced transcripts and the mature species with spliced transcripts. We use the nascent/mature nomenclature to simplify notation and emphasize that this identification is natural for scRNA-seq data, but not mandatory in general.

Formalizing a model in terms of the CME requires specifying the precise mechanistic meaning of *ρ_cg_* and ℓ*_c_*. Previous reports equivocate regarding the latter [11], appealing either to cell-wide effects on the biology (in the spirit of [24, 25]) or technical variability in the sequencing process (in the spirit of [33]). For completeness, we treat both cases.

Below, we present the theoretical results, including the biophysical models, the functional forms of bivariate distributions consistent with the standard *scVI* models, and the consequences of introducing further assumptions. The full derivations are given in Section S1.

#### 4.3.1 *Constitutive*: The Poisson model and its mechanistic basis

The Poisson generative model can be recapitulated by the following schema:

(6) $$\emptyset \overset{k}{\to }𝒩\overset{\beta }{\to }ℳ\overset{\gamma }{\to }\emptyset ,$$

where *k* is a constant transcription rate. This process converges to the bivariate Poisson stationary distribution, with the following likelihood:

(7) $$P(n,m;\mu_{N},\mu_{M})=P_{\text{Poiss}}(n;\mu_{N})P_{\text{Poiss}}(m,\mu_{M}),$$

where *μ_N_* = *k/β* and *μ_M_* = *k*/γ. If we suppose each gene’s *β* and γ are constant across cell types, the likelihoods involve a single compositional parameter *ρ_cg_*, such that

(8) $$\mu_{N}=\frac{\gamma_{g}}{\beta_{g}}\rho_{cg}\ell_{c}\;\mu_{M}=\rho_{cg}\ell_{c},$$

where $\gamma_{g}∕\beta_{g}\in R^{+}$ is a gene-specific parameter that can be fit or naïvely estimated by the ratio of the unspliced and spliced averages. On the other hand, if the downstream processes’ kinetics can also change between cell types, we must use two compositional parameters:

(9) $$\mu_{N}=\rho_{cg}^{\left(N\right)}\ell_{c}\;\mu_{M}=\rho_{cg}^{\left(M\right)}\ell_{c}.$$

We refer to this model as “Poisson,” reflecting its functional form, or “constitutive,” reflecting its biophysical basis.

#### 4.3.2 *Extrinsic*: The negative binomial model and a possible mixture basis

The negative binomial generative model can be recapitulated by the following schema:

(10) $$\emptyset \overset{k\;∼\;K}{\to }𝒩\overset{\beta }{\to }ℳ\overset{\gamma }{\to }\emptyset ,$$

where *k* is the transcription rate, a realization of *K*, a gamma random variable with shape *α*, scale *η*, and mean ⟨*K*⟩ = *αη*. This process converges to the bivariate negative binomial (BVNB) stationary distribution, with the following likelihood:

(11) $$P_{\text{extrinsic}}(n,m;\alpha ,\mu_{N},\mu_{M})=\frac{\Gamma (\alpha +n+m)}{n!m!\Gamma (\alpha )}{\left(\frac{1}{\alpha +\mu_{N}+\mu_{M}}\right)}^{\alpha +n+m}\alpha^{\alpha }\mu_{N}^{n}\mu_{M}^{m},$$

where *μ_N_* = ⟨*K*⟩/*β* and *μ_M_* = ⟨*K*⟩/γ. If we suppose that cell type differences only involve changes in the transcription rate scaling factor *η*, with constant *α*, *β*, and γ, the likelihoods involve a single compositional parameter *ρ_cg_*. The mean parameters are identical to Equation 8, with an analogous parameter γ*_g_*/*β_g_*, as well as a gene-specific shape parameter *α_g_*. On the other hand, if the downstream processes’ kinetics can also change between cell types, we must use two compositional parameters, as in Equation 9.

We refer to this model as “extrinsic” to reflect its biophysical basis in extrinsically stochastic rates of transcriptional initiation.

#### 4.3.3 *Bursty*: The negative binomial model and a possible bursty basis

The negative binomial generative model may be recapitulated by the alternative schema [22]:

(12) $$\emptyset \overset{k}{\to }B\times 𝒩\overset{\beta }{\to }ℳ\overset{\gamma }{\to }\emptyset ,$$

where *k* is the burst frequency and *B* is a geometric random variable with mean *b* (Equation 3). This system converges to the following stationary distribution:

(13) $$P_{\text{bursty}}(n,m;\alpha ,\mu_{N},\mu_{M})=P_{\text{NB}}(n;\alpha ,\mu_{N})P(m|n;\alpha ,\mu_{N},\mu_{M}),$$

where *μ_N_* = *kb/β*, *μ_M_* = *kb*/γ, and *α* is arbitrarily set to *k/β* for simplicity.

Although the nascent marginal is known to be negative binomial, the joint *P*(*n,m*) and conditional *P*(*m*∣*n*) distributions are not available in closed form. For a given set of parameters, the joint distribution can be approximated over a finite microstate domain *n*, *m* ∈ [0, ß*_N_* – 1] × [0, ß*_M_* – 1], with total *s*tate *s*pace size ß*_N_* ß*_M_*. This approach is occasionally useful, if intensive, for evaluating the likelihoods of many independent and identically distributed samples. The numerical procedure entails using quadrature to calculate values of the generating function on the complex unit sphere, then performing a Fourier inversion to obtain a probability distribution [22]. However, this strategy is inefficient in the variational autoencoder framework, where each observation is associated with a distinct set of parameters. Furthermore, it is likely incompatible with automatic differentiation.

In [23], we demonstrated that the numerical approach can be simplified by approximating *P*(*m*∣*n*) with a learned mixture of negative binomial distributions: the weights are given by the outputs of a neural network, whereas the negative binomial bases are constructed analytically. The neural network is trained on the outputs of the generating function procedure. Although the generative model does not have a simple closed-form expression, it is represented by a partially neural, pre-trained function that is *a priori* compatible with the VAE.

If we suppose cell type differences only involve changes in the burst size *b*, with constant *k, β* and γ, we use Equation 13 to evaluate likelihoods. These likelihoods involve a single compositional parameter *ρ_cg_*, with mean parameters identical to Equation 8, with an analogous parameter γ*_g_*/*β_g_*, as well as a gene-specific shape parameter *α_g_*. On the other hand, if kinetics of the degradation process can also change between cell types, we must use two compositional parameters, as in Equation 9. There is no admissible way to allow modulation in the burst frequency.

We refer to this model as “bursty,” reflecting its biophysical basis.

### 4.4 *biVI* modifications

We built modifications upon *scVI* version 0.18.0 [34]. The *scVI* framework already supports the constitutive model. By setting conditional likelihood to “poisson,” no modification of *scVI* architecture is necessary. The conditional likelihood is the product of two Poisson distributions (Equation 7).

For the extrinsic and bursty models, mean parameters for nascent and mature counts, *μ_N_* and *μ_M_*, and a single shape parameter *α* are necessary. The default *scVI* architecture returns two independent parameters for nascent and mature counts of the same gene. *biVI* thus modifies the *scVI* architecture to update vectors $\alpha \in R_{\geq 0}^{N_{G}}$ rather than $\alpha \in R_{\geq 0}^{2N_{G}}$, where *N_G_* is the number of genes. For the extrinsic model, the conditional likelihood is set to the extrinsic likelihood *P*_extrinsic_(*n*, *m*; *α*, *μ_N_*, *μ_M_*) (Equation 11). For the bursty model, the conditional likelihood is set to the bursty likelihood *P*_bursty_(*n*, *m*; *α*, *μ_N_*, *μ_M_*) (Equation 13).

### 4.5 Simulated data

To validate our implementation of *biVI*, and to understand potential pitfalls inherited from the standard *scVI* autoencoder workflow, we generated ground truth data by simulation. Simulated ground truth is limited in important ways, as it omits known and unknown sources of biological and technical variability. However, to begin to understand the limitations of integrating descriptive autoencoders with mechanistic models, it is helpful to have simple and well-understood simulated datasets. By judiciously choosing simulation scenarios, we can characterize the relative and absolute accuracy of the procedures under ideal-case conditions, which describe a natural upper bound on their performance.

The simulation procedures are described in Section S2. For each of the three models (constitutive, extrinsic, and bursty), we simulated a data set of nascent and mature RNA counts for 2,000 genes across 10,000 cells, spread across 20 cell types. The cell types were distinguished by low-magnitude variation in all genes’ parameters, as well as higher-magnitude upregulation of expression in a small set of cell type-specific marker genes. In the extrinsic noise model, upregulation was effected by increasing the transcription rate scale *η*. In the analogous constitutive case, it was effected by increasing the transcription rate *k*. In the bursty model, it was effected by increasing the burst size *b*.

#### 4.5.1 Fitting simulated data

We fit the three simulated data sets using *biVI* with the three generative physical models, as well as standard *scVI* with the negative binomial distribution as its conditional likelihood. All models were trained on 7,200 cells with 800 validation cells for 400 epochs with a learning rate of 0.001. The encoder and decoder networks consisted of 3 layers with 128 nodes each, with a latent space of dimension 10. As in standard *scVI*, a standard normal prior was used for the latent space.

#### 4.5.2 Performance metrics on simulated data

Performance of the trained models was tested on 2,000 held-out cells for each simulated data set. In order to characterize the absolute reconstruction of ground truth cell types, the mean squared error (MSE) between *biVI* inferred means, *μ_N_* and *μ_M_*, and ground truth simulated means was calculated for each cell. Furthermore, we assessed how well different models reduced cells of the same cell type to similar latent spaces while maintaining good separation of cells in different cell types. To compare clustering accuracy for different models, we applied several metrics to the latent spaces obtained for testing data, using simulated ground truth cell type as cluster assignment. We calculated average intra-cluster distance (ICD), or the Euclidean distance between each cell’s latent representation and the mean of its assigned cluster in the latent space averaged over all cells. We also calculated average intra-cluster variance (ICV), or the variance of ICDs for each cell in a cluster averaged over all clusters. Inter-cluster distance was also calculated: distance between cluster means averaged over all pair-wise clusters (excluding the cluster’s distance to its own mean). Finally, scaled inter-cluster distance, or inter-cluster distance divided by average intra-cluster distance, was found. We also report nearest neighbor percentages (Figures S3, S4, S5), or the percent of the *n_k_* nearest neighbors in the same cell type as each cell, where *n_k_* is the number of cells in the given cell’s cell type. A quantitative description of clustering metrics is provided in Section S4. Broadly speaking, these metrics characterize the methods’ utility for discovering discrete cell populations using typical clustering algorithms, which perform best when low-dimensional clusters are relatively condensed, homogeneous, and reflect the high-dimensional structure.

#### 4.5.3 Reconstructing gene distributions

A simulated cell type is defined by cell type-specific parameters for each gene, *θ_kg_*, where *k* indexes over cell types, *g* indexes over genes, and *θ* contains the model parameters. Each cell in a cell type has identical parameters. *biVI* and *scVI*, however, infer parameters for every cell and gene: *θ_cg_*, where *c* indexes over cells and *g* indexes over genes. To compare reconstructed distributions to simulated ground truth distributions for a given gene of a given cell type, we first select all cells of that cell type. Next, we define the distribution under a given model for each cell using *biVI* or *scVI* inferred parameters. We average over the cell-specific probabilities for a given gene to produce a cell-type distribution for that gene:

(14) $${\hat{P}}_{\kappa g}(n,m)=\frac{1}{n_{\kappa }}\sum_{c_{\kappa }=1}^{n_{\kappa }}P(n,m;\theta_{c_{\kappa }g}),$$

where *n_k_* is the total number of cells in cell type *k*, and *c_k_* indexes over all cells in that cell type. This identity follows immediately from defining the cell type’s distribution as the mixture of the distributions of its constituent cells. In the case of *biVI*, we plug in Equation 7, 11, or 13 for *P*(*n*, *m*; *θ_ckg_*). In the case of *scVI*, we use a product of two independent negative binomial laws:

(15) $$P(n,m;\theta_{c_{\kappa }g})=P_{\text{NB}}(n;\alpha_{g}^{N},\mu_{N})P_{\text{NB}}(m;\alpha_{g}^{M},\mu_{M}),$$

where *μ_N_* and *μ_M_* are cell- and gene-specific, whereas *α^N^* and *α^M^* are fit separately and take on different values (Section 4.4). For simplicity, this comparison omits uncertainty associated with *θ_cg_*, which is formally inherited from the uncertainty in the latent representation *z* for each cell *c*.

#### 4.5.4 Kullback-Leibler divergence between simulated and reconstructed distributions

We compare *biVI* and *scVI*’s ability to accurately reconstruct ground truth gene distributions by calculating the truncated Kullback-Leibler divergence (KLD) between ground truth simulated distributions and reconstructed distributions for all genes in all cell types. We reconstruct gene and cell-type specific *biVI* and *scVI* distributions $\hat{P}(n,m)$ as described above in Section 4.5.3. We also calculate true distributions *Q_kg_*(*n, m*; *θ_kg_*) using the gene and cell-type specific ground truth simulated parameters *θ_kg_* (parameter generation and sampling are described in Section S2). We calculate both true and reconstructed model (*biVI* or *scVI*) distributions over a 50 by 50 grid of nascent and mature values, then normalize both to sum to 1.0 over the grid by dividing all probabilities by their sum over the grid. Normalization ensures that KL divergences are well-defined. We then calculate the KL divergence over the restricted grid:

(16) $$\text{KLD}=\sum_{m=0}^{49}\sum_{n=0}^{49}{\hat{P}}_{\kappa g}(n,m)\times \log\;\frac{{\hat{P}}_{\kappa g}(n,m)}{Q_{\kappa g}(n,m;\theta_{\kappa g})}.$$

### 4.6 Connecting the models to transcriptome data

Thus far, we have used “nascent” and “mature” as shorthands for the discrete species in a two-stage model of RNA processing. In other words, Equation 5 is axiomatic for this nomenclature. We have gone one step further and named the rate of conversion *β* the *splicing rate*, explicitly identifying the nascent species with unspliced RNA and the mature species with spliced RNA. This identification is a modeling decision that elides the considerable simplification of biological complexities. In the current section, we elaborate on the assumptions.

In the field of microbiology, “nascent” RNA is often, but not always, used to characterize the mRNA molecules in the process of synthesis, associated to a DNA strand via an RNA polymerase complex [35-38]. In this framework, the “mature” transcriptome is simply the complement of the nascent transcriptome, i.e., all molecules that are not chemically associated to a DNA strand. Therefore, the canonical definition of “nascent” RNA is equivalent to *transcribing* RNA, which is a polymeric structure with a particular sequence.

Transcribing RNA can be observed directly through electron micrography [37]. However, more typically, it is investigated through more or less direct experimental proxies that can be scaled to many genes and cells at a time. In the single-cell fluorescence subfield, DNA or membrane staining can be used to identify bright spots localized to the nucleus, which is treated as signal from RNA at the transcription site [29, 39]; this signal may include contributions from RNA incidentally, or mechanistically, retained at a DNA locus [36]. In this strategy, “nascent” molecules are DNA-associated. Alternatively, and more commonly, transcribing molecules have been studied by using probes targeted to the 5′ and 3′ regions [40-43], or to intronic and exonic regions [44-47]. In this strategy, “nascent” molecules contain a particular region, either synthesized earlier or removed later in the RNA life-cycle.

The use of intron data as a proxy for active transcription is reminiscent of, but distinct from sequence census [48] strategies that directly study RNA sequences. These strategies, in turn, typically use chemical methods to enrich for newly transcribed RNA. For example, Reimer et al. isolate chromatin, then deplete sequences that have been post-transcriptionally poly(A) tailed [49]. Analogously, Drexler et al. use 4-thiouridine (4sU) labeling to enrich for newly synthesized molecules [50]. These approaches may produce conflicting results; for example, introns may be rich both in poly(A) handles [1] and 4sU targets [49], giving rise to obscure technical effects. Therefore, these “processed” or “temporally labeled” proxies are coarsely representative of transcriptional dynamics, and their quantitative interpretability is unclear as of yet.

The sequence content may be used more directly, by conceding that DNA association or localization are not easily accessible by sequence census methods, and treating splicing *per se*. This approach has a fairly long history. Intronic quantification has been used to characterize transcriptional mechanisms in microarray datasets [51], and to characterize differentiation programs in RNA sequencing [52,53]. In single-cell RNA sequencing, intronic content has been leveraged to identify transient behaviors from snapshot data [1], albeit with some outstanding theoretical concerns and caveats [54]. Briefly, it is, in principle, possible to coarsely classify molecules with intronic content as “unspliced” or “pre-mRNA” and aggregate all others as “spliced,” “mature,” or simply “mRNA.” By applying this binary classification, and defining a simple first-order model of splicing, data may be successfully fit. The quantification of transcripts so classified is a relatively straightforward genomic alignment problem. The multiple available implementations [1, 2, 55, 56] tend to disagree on the appropriate assignment of ambiguous sequencing reads [54,56], obscuring a more fundamental problem: the binary classification is mechanistically limited [50,57-59], and it is likely that detailed splicing graph models will be necessary in the future [31,54]. The focus on sequence is yet another step removed from the transcriptional dynamics, particularly since some of the splicing processes occur after transcriptional elongation is complete [60]. However, in spite of its limitations and assumptions, the simple Markovian two-stage model has been successful in the past [61,62]. Mechanistic evidence does not suggest that, e.g., deterministic delayed elongation is necessary to represent “unspliced” distributions [63]: under this model, we appear to be able to treat splicing and degradation as Markovian, without representing the elongation process at all, and obtain reasonable fits to the data. Somewhat surprisingly [64], the geometric-Poisson distribution, which describes bursty transcription coupled to deterministic elongation [65], is a particularly poor fit for unspliced RNA counts [63].

Adding yet more complexity to the modeling, “mature” – whether “off-template,” “spliced,” or “processed” – molecules are not immediately available for degradation; first, the process of nuclear export must take place. Studies that presuppose access to imaging data tend to model it explicitly [22, 29, 66-68]. However, this approach has not been applied in sequencing assays, as current technologies do not distinguish nuclear and cytoplasmic molecules. Furthermore, comparisons of paired single-cell and single-nucleus datasets are hampered by the limited characterization of the noise sources in the latter technology. Yet again, we have generally found that omitting this effect in single-cell data produces acceptable fits [61-63].

Pending the development of more sophisticated sequencing and alignment technologies, as well as the implementation of tractable models of biology, the data exploration portion of our study focuses on the “spliced” and “unspliced” matrices generated by *kallisto* ∣*bustools* [2]. This choice is a compromise, and we adopt it after considering the following factors:

- Availability of quantification workflows: spliced and unspliced matrices are straightforward to generate.
- Model tractability: the two-stage models can be evaluated [23]; more sophisticated models require new algorithms to be integrated with variational autoencoders.
- The scope of sequencing data: single-cell protocols do not yet give access to sub-cellular information, so inference of elongation or nuclear retention dynamics is acutely underspecified.
- Self-consistency and compatibility: *a priori*, we seek to recapitulate the data types (spliced RNA counts) and distributions (negative binomial and Poisson) already implemented in *scVI*, leaving improvements to further investigation.

We use the terms “nascent” and “mature” to identify the unspliced and spliced RNA matrices. This choice of nomenclature is deliberate. Although it somewhat conflicts with the established microbiology literature, this terminology is intended to emphasize the models’ generality, both here and elsewhere [9, 23, 69]. The two-stage Markovian process is axiomatic. The specific identities assigned to the mathematical objects may range beyond counts identified by sequence census methods. They may represent the discretized and subtracted intensities of 3’, 5’, intron, or exon fluorescent probes, the counts of molecules within and outside the nuclear envelope, or polymerase counts obtained by micrography. Therefore, the terminology should be taken in the sense used for similar non-delayed models in [64, 66, 70, 71].

### 4.7 Preprocessing Allen data

Raw 10x v3 single-cell data were originally generated by the Allen Institute for Brain Science [26]. The raw reads in FASTQ format [72] and cluster metadata [73] were obtained from the NeMO Archive. We selected mouse library B08 (donor ID 457911) for analysis.

To obtain spliced and unspliced counts, we first obtained the pre-built mm10 mouse genome released by 10x Genomics (https://support.10xgenomics.com/single-cell-gene-expression/software/downloads/latest, version 2020-A). We used *kallisto*∣*bustools* 0.26.0 [2] to build an intronic/exonic reference (kb ref with the option --lamanno). Next, we pseudoaligned the reads to this reference (kb count with the option --lamanno). We used the outputs produced by the standard *bustools* filter. This filter was relatively permissive: all (8,424) barcodes given cell type annotations in the Allen metadata were present in the output count matrix (10,975 barcodes).

Based on previous clustering results, we selected cells that were given cell type annotations, and omitted “low quality” or “doublet” barcodes [26], for a total of 6,418 cells. Although any choice to retain or omit cells from analysis is arbitrary, our work models the generating process that produced cells’ nascent and mature counts by presupposing each barcode corresponds to a single cell. Therefore, we propose that cells identified as low-quality (empty cells) or as doublets (two cells measured in one observation) [26] have a fundamentally different data-generating process than individual single cells, and therefore remove them before fitting VAE models. However, we stress that the stochastic nature of transcription and sequencing, the intrinsic uncertainties associated with read alignment, and the numerical compromises made in clustering large datasets mean that previous annotations are not “perfect,” merely a reasonable starting point for comparing alternative methods.

We used Scanpy [74] to restrict our analysis to several thousand variable genes, which presumably reflect the cell type signatures of interest. The mature count matrix for the 6,418 retained cells was normalized to sum to 10,000 counts per cell, then transformed with log1p. The top 2,000 most highly variable genes were identified using scanpy.pp.highly_variable_genes with minimum mean of 0.0125, maximum mean of 3, and minimum dispersion of 0.5 [74].

### 4.8 Fitting Allen data

We applied *biVI* with the three generative models (bursty, constitutive, and extrinsic) and *scVI* with negative binomial likelihoods to the count matrices obtained by the filtering procedures outlined above. 4,622 cells were used for training with 513 validation cells, and 1,283 cells were held out for testing performance. All models were trained for 400 epochs with a learning rate of 0.001. Encoders and decoder consisted of 3 layers of 128 nodes, and each model employed a latent dimension of 10.

### 4.9 Differential parameter values and gene expression

After fitting the VAE models, we sought to identify meaningful statistical differences that distinguish cell types. In order to avoid instabilities, we excluded cell subclasses “L6 IT Car3,” “L5 ET,” “VLMC,” and “SMC” from this analysis, as they contained fewer than ten annotated cells, necessitating more sophisticated statistical models that take into account their small sample sizes. The following analysis thus considers 6,398 cells in 16 unique subclasses. We only computed differential expression metrics under the bursty model.

Differential parameter values and gene expression were tested for each assigned subclass label (as annotated in [26]) versus all others using two-sided *t*-tests implemented in scipy.stats.ttest_ind [75] under the null hypothesis that there was no difference in the average of the tested values. We applied Bonferroni corrections for the two-tailed test, the 16 subclass labels, and the 2,000 genes: values were considered significantly differential if the *p*-value under the *t*-test was below 0.05 × (2 × 16 × 2, 000)^−1^ = 7.813 × 10^−7^. To assign significance, we further required that the magnitude of log_2_ fold change to be greater than 1, i.e., the arithmetic mean of the tested value was doubled or halved in one cell subclass versus the rest. We adopted this effect size threshold due to the relatively large number of cells and corresponding prevalence of genes with significant p-values.

The *t*-tests were performed on normalized burst size and means, i.e., the inferred burst size or inferred means divided by observed sequencing depth for that cell. The observed sequencing depth of a cell ℓ*_c_* was set to the sum of all observed counts, nascent and mature, in that cell. Normalization was thus done to ensure that identified differences in parameters was not due to sequencing depth but was biologically meaningful. Relative degradation rate is independent of sequencing depth: t-tests were performed directly on inferred relative degradation rates.

As discussed in Section 4.4, *scVI* fits the cell-specific parameters $\rho_{cg}^{\left(N\right)}$ and $\rho_{cg}^{\left(M\right)}$, as well as gene-specific parameters $\alpha_{g}^{N}$ and $\alpha_{g}^{M}$. To compute effect sizes and *p*-values, we simply compared the distributions of *ρ*_*cg*_ in distinct cell populations, using $\rho_{cg}^{\left(N\right)}$ for differences in nascent expression and $\rho_{cg}^{\left(M\right)}$ for differences in mature expression.

As discussed in Section S1.3 *biVI* fits $\rho_{cg}^{\left(N\right)}$, $\rho_{cg}^{\left(M\right)}$ and *α_g_* under the bursty generative model. To calculate parameters, we use the definitions outlined in the section, enforcing *α_g_* = *k_g_/β_g_*. This yields the following relationships:

(17) $$\beta_{g}=\frac{k_{g}}{\alpha_{g}},\;\gamma_{z}=\frac{\rho_{cg}^{\left(N\right)}}{\rho_{cg}^{\left(M\right)}}\frac{k_{g}}{\alpha_{g}},\;b_{z}=\frac{\rho_{cg}^{\left(N\right)}}{\alpha_{g}}C,$$

where *C* is a large multiplicative constant shared across cells and genes. We set *k_g_* to unity with no loss of generality at steady state. Finally, we compare the inferred values of *γ_z_*/*k_g_* and *b_z_*/*C* to identify differentially expressed genes.

## Supplementary Material

Supplement 1

## Data Availability

Simulated datasets, simulated parameters used to generate them, and Allen dataset B08 and its associated metadata are available in the Zenodo package 7497222. All analysis scripts and notebooks are available at https://github.com/pachterlab/CGCCP_2023. The repository also contains a Google Colaboratory demonstration notebook applying the methods to a small human blood cell dataset.
