# Supplementary material for: Mechanistic modeling with a variational autoencoder for multimodal single-cell RNA sequencing data
Source: bioRxiv. 2023 Jan 14:2023.01.13.523995. Preprint. [Version 1] doi: 10.1101/2023.01.13.523995 (PMC9882246; doi:10.1101/2023.01.13.523995)
Supplement: Supplementary file 1 [file media-1.pdf]

Mechanistic modeling with a variational autoencoder for multimodal single-cell RNA sequencing data
Supplementary Material

Maria Carilli[1,*], Gennady Gorin[2,*], Yongin Choi[3,4], Tara Chari[1], and Lior Pachter[1,5,**]

[1]Division of Biology and Biological Engineering, California Institute of Technology
[2]Division of Chemistry and Chemical Engineering, California Institute of Technology
[3]Biomedical Engineering Graduate Group, University of California, Davis
[4]Genome Center, University of California, Davis
[5]Department of Computing and Mathematical Sciences, California Institute of Technology
[*]These two authors contributed equally to this work.
[**]lpachter@caltech.edu

January 13, 2023

S1 Derivations

As discussed in Section 4.3, our models encode a series of single-molecule stochastic processes.

S1.1 The Poisson model and its constitutive extensions

The simplest standard *scVI* generative model is Poisson (Equation 1), where $\mu = \mu_{cg}$ is cell- and gene-specific. Furthermore, $\mu_{cg} = \rho_{cg}(z)\ell_c$ encodes dependence on the latent encoding z of cell c through the compositional gene abundance ρ_{cg} (such that $\sum_g \rho_{cg} = 1$), as well as the genome-wide "cell size" scaling factor ℓ_c.

The following model produces a Poisson stationary distribution of $\mathcal{X}$ [76]:

$$\varnothing \xrightarrow{k} \mathcal{X} \xrightarrow{\gamma} \varnothing. \tag{18}$$

This is the standard model of constitutive, or unregulated, transcription [77] at a single gene locus, with mean $\mu = k/\gamma$.

S1.1.1 "Cell size" as biological variation

The parameter k is the transcription rate. In physical terms, it typically represents the rate of RNA polymerase (RNAP) recruitment at the gene locus. The rate of recruitment may be modeled by a simple mass action model:

$$k_{cg} = k_{cg,init}[\text{RNAP}]_c. \tag{19}$$

Thus, the concentration of RNAP is cell-specific, and controls the transcription rate for all genes. On the other hand, the initiation rate differs between cells (e.g., due to different levels of chromatin accessibility) as well as different genes (e.g., due to differences in binding kinetics between promoter motifs). We may choose the units of concentration to yield $[\text{RNAP}]_c \sim \mathcal{O}(1)$. This formulation implies:

$$\mu_{cg} = \frac{k_{cg}}{\gamma_g} = \frac{k_{cg,init}}{\gamma_g}[\text{RNAP}]_c = \frac{k_{z,init}}{\gamma_g}[\text{RNAP}]_c, \tag{20}$$

where we assume γ is constant across different cells.

The first factor encodes transcriptional modulation due to the specific gene identity and location in latent space, whereas the second factor encodes transcriptional modulation due to "cell size" effects. If we include an arbitrary, but large universal scaling factor C (on the order of 10^4), we obtain

$$\mu_{cg} = \frac{k_{z,init}}{C\gamma_g}C[\text{RNAP}]_c, \tag{21}$$

and can *define* the *scVI* factors as:

$$\begin{aligned} \rho_{cg} &:= \frac{k_{z,init}}{C\gamma_g}, \\ \ell_c &:= C[\text{RNAP}]_c, \end{aligned} \tag{22}$$

which accords with the premise that the values of ρ_{cg} are near zero, whereas those of ℓ_c are quite large and comparable to the cells' total UMI counts.

Next, we write down the two-species system:

$$\varnothing \xrightarrow{k} \mathcal{N} \xrightarrow{\beta} \mathcal{M} \xrightarrow{\gamma} \varnothing, \tag{23}$$

which induces the bivariate Poisson stationary distribution:

$$P(n, m; \mu_N, \mu_M) = P_{\text{Poiss}}(n; \mu_N) P_{\text{Poiss}}(m; \mu_M), \tag{24}$$

where $\mu_N = k/\beta$ and $\mu_M = k/\gamma$. We maintain the assumptions of z and "cell size" dependence through k, and impose the assumption that both β and γ are gene-specific and constant. This yields:

$$\begin{aligned} \mu_{cg}^{(N)} &= \frac{k_{z,init}}{C\beta_g} C[\text{RNAP}]_c = \frac{\gamma_g}{\beta_g} \rho_{cg} \ell_c, \\ \mu_{cg}^{(M)} &= \frac{k_{z,init}}{C\gamma_g} C[\text{RNAP}]_c = \rho_{cg} \ell_c, \end{aligned} \tag{25}$$

where γ_g/β_g may be fit or estimated from the plug-in method of moments estimate $\overline{N}_g/\overline{M}_g$. On the other hand, if we suppose that γ and β can differ between cell types, we are forced to define two distinct ρ parameters:

$$\begin{aligned} \mu_{cg}^{(N)} &= \rho_{cg}^{(N)} \ell_c \text{ s.t. } \rho_{cg}^{(N)} := \frac{k_{z,init}}{C\beta_z}, \\ \mu_{cg}^{(M)} &= \rho_{cg}^{(M)} \ell_c \text{ s.t. } \rho_{cg}^{(M)} := \frac{k_{z,init}}{C\gamma_z}. \end{aligned} \tag{26}$$

In sum, if we assume each gene's splicing and degradation rates are constant across all cells, we use Equation 24 to evaluate likelihoods, with $\mu_N = \frac{\gamma_g}{\beta_g} \rho_{cg} \ell_c$ and $\mu_M = \rho_{cg} \ell_c$. On the other hand, if we relax this assumption, we use the same equation, with $\mu_N = \rho_{cg}^{(N)} \ell_c$ and $\mu_M = \rho_{cg}^{(M)} \ell_c$.

S1.1.2 "Cell size" as technical variation

We can recapitulate the same quantitative model by describing ℓ_c as reflective of a technical phenomenon. First, we replace $\mathcal{X}$ with $\mathcal{X}_b$, the *biological* molecules. Next, we impose the hypothesis that each molecule of $\mathcal{X}_b$ has probability p of actually being captured, amplified, and sequenced during the experiment. The sequencing process induces a Poisson stationary distribution of observed counts $\mathcal{X}$, with mean $\mu = kp/\gamma$. This model can be obtained from a model of conversion $\mathcal{X}_b \to \mathcal{X}$ or in the low-rate limit of a model of catalytic production $\mathcal{X}_b \to \mathcal{X}_b + \mathcal{X}$ [61].

Therefore, we can parameterize a simple generative model of imperfect capture:

$$\mu_{cg} = \frac{k_{cg}}{\gamma_{cg}} p_c = \mu_{cg}^b p_c, \tag{27}$$

i.e., downsampling amounts to rescaling the expectation of the *b*iological quantity. The assumption that p_c does not vary between genes reflects a specific class of hypotheses, e.g., poly(A) tails being chemically similar in a sequencing experiment. This model may also reflect more generic

descriptions of variation, as the factors of μ_{cg} are not independently identifiable. For example, if $p_{cg} = p_c\xi_g$ for some coefficient ξ_g of moderate magnitude, we merely find that μ^b_{cg} is rescaled by ξ_g.

If we introduce the scaling factor C, we obtain

$$\mu_{cg} = \frac{\mu^b_{cg}}{C} C p_c. \tag{28}$$

Much as before, if C is relatively large, we may define the variables as:

$$\begin{aligned} \rho_{cg} &:= \frac{\mu^b_{cg}}{C}, \\ \ell_c &:= C p_c. \end{aligned} \tag{29}$$

In the two-species case (Equation 23), the same identities hold [62]. The bivariate law is given by Equation 24 with $\mu_N = \mu^b_N p_c$ and $\mu_M = \mu^b_M p_c$. As above, the assumption of a common p_c is not mandatory, but the functional form can be derived by proposing $p^{(N)}_{cg} = \xi^{(N)}_{cg} p_c$ and $p^{(M)}_{cg} = \xi^{(M)}_{cg} p_c$, followed by rescaling the biological expectations. Thus, if β and γ can differ, we find:

$$\begin{aligned} \mu^{(N)}_{cg} &= \frac{k_z}{\beta_z}\ell_c = \rho^{(N)}_{cg}\ell_c, \\ \mu^{(M)}_{cg} &= \frac{k_z}{\gamma_z}\ell_c = \rho^{(M)}_{cg}\ell_c. \end{aligned} \tag{30}$$

This distinct set of assumptions recapitulates the functional form in the previous section. To obtain this solution, we used a convenient property by which a Bernoulli-subsampled Poisson distribution remains Poisson.

S1.2 The negative binomial model and its mixture extensions

The *scVI* negative binomial generative model is based on (Equation 2), where $\mu = \mu_{cg}$ is defined as above, whereas $\alpha = \alpha_g$ is the shape or "inverse dispersion" parameter, presumed to be gene-specific.

The following model produces a negative binomial stationary distribution of $\mathcal{X}$:

$$\varnothing \xrightarrow{k \sim K} \mathcal{X} \xrightarrow{\gamma} \varnothing, \tag{31}$$

where the transcription rate k is drawn from a gamma distribution with shape α, scale η, and mean $\langle K \rangle = \alpha\eta$. This is the mixture extension of the constitutive model, which encodes variability in the transcription rate [24, 25], potentially due to an unspecified, but slow, stochastic process [9]; the mean of this distribution is $\langle K \rangle/\gamma$.

S1.2.1 "Cell size" as biological variation

To include dependence on z and the "cell size," while maintaining the assumption of a constant shape parameter, we can generalize the model in Section S1.1.1. First, we note that η, the gene-specific scale parameter that governs the frequency of transcriptional events, must be proportional to the concentration term [RNAP] [9]. By choosing the appropriate units and defining $\eta_{cg} =$

$k_{z,init}[\text{RNAP}]_c$, we obtain

$$\begin{aligned}\mu_{cg} &= \frac{\alpha_g k_{z,init}}{C\gamma_g} C[\text{RNAP}]_c,\\ \rho_{cg} &:= \frac{\alpha_g k_{z,init}}{C\gamma_g},\\ \ell_c &:= C[\text{RNAP}]_c,\end{aligned} \tag{32}$$

which is consistent with the Poisson result. Next, we write down the two-species system:

$$\varnothing \xrightarrow{k \sim K} \mathcal{N} \xrightarrow{\beta} \mathcal{M} \xrightarrow{\gamma} \varnothing, \tag{33}$$

which induces the bivariate negative binomial stationary distribution [78]:

$$P(n, m; \alpha, \mu_N, \mu_M) = \frac{\Gamma(\alpha + n + m)}{n!m!\Gamma(\alpha)} \left(\frac{\alpha}{\alpha + \mu_N + \mu_M}\right)^{\alpha} \left(\frac{\mu_N}{\alpha + \mu_N + \mu_M}\right)^{n} \left(\frac{\mu_M}{\alpha + \mu_N + \mu_M}\right)^{m}. \tag{34}$$

As in the generic case, $\mu_N = \langle K \rangle / \beta$ and $\mu_M = \langle K \rangle / \gamma$. We maintain the assumptions of z and "cell size" dependence through k, and impose the assumption that both β and γ are gene-specific and constant. This yields:

$$\begin{aligned}\mu_{cg}^{(N)} &= \frac{\alpha_g k_{z,init}}{C\beta_g} C[\text{RNAP}]_c = \frac{\gamma_g}{\beta_g} \rho_{cg} \ell_c,\\ \mu_{cg}^{(M)} &= \frac{\alpha_g k_{z,init}}{C\gamma_g} C[\text{RNAP}]_c = \rho_{cg} \ell_c,\end{aligned} \tag{35}$$

where γ_g/β_g may be fit or estimated from the plug-in method of moments estimate $\overline{N}_g/\overline{M}_g$. As before, if we suppose that γ and β can differ between cell types, we are forced to define two distinct ρ parameters:

$$\begin{aligned}\mu_{cg}^{(N)} &= \rho_{cg}^{(N)} \ell_c \text{ s.t. } \rho_{cg}^{(N)} := \frac{\alpha_g k_{z,init}}{C\beta_g},\\ \mu_{cg}^{(M)} &= \rho_{cg}^{(M)} \ell_c \text{ s.t. } \rho_{cg}^{(M)} := \frac{\alpha_g k_{z,init}}{C\gamma_g}.\end{aligned} \tag{36}$$

In sum, if we assume each gene's splicing and degradation rates are constant across all cells, we use Equation 34 to evaluate likelihoods, with $\mu_N = \frac{\gamma_g}{\beta_g} \rho_{cg} \ell_c$, $\mu_M = \rho_{cg} \ell_c$, and a shared fit parameter α_g. On the other hand, if we relax this assumption, we use the same equation, with $\mu_N = \rho_{cg}^{(N)} \ell_c$ and $\mu_M = \rho_{cg}^{(M)} \ell_c$.

S1.2.2 "Cell size" as technical variation

As in Section S1.1.2, we may propose a model of imperfect capture that recapitulates the functional form in *scVI*. Under Bernoulli sampling, the univariate negative binomial model stays negative

binomial with the same shape parameter:

$$\begin{aligned}\mu_{cg} &= \frac{\langle K\rangle_{cg}}{\gamma_{cg}} p_c = \frac{\alpha_g \eta_z}{\gamma_z} p_c := \mu_{cg}^b p_c \\ &= \frac{\alpha_g \eta_z}{C\gamma_z} C p_c,\end{aligned} \tag{37}$$

i.e., downsampling amounts to rescaling η_z/γ_z. The *scVI* variables can be interpreted as follows:

$$\begin{aligned}\rho_{cg} &:= \frac{\alpha_g \eta_z}{C\gamma_z}, \\ \ell_c &:= C p_c.\end{aligned} \tag{38}$$

In the two-species case, the same identities hold [62]. The bivariate law is given by Equation 34 with $\mu_N = \mu_N^b p_c$ and $\mu_M = \mu_M^b p_c$. As above, the assumption of a common p_c is not mandatory. Thus, if β and γ can differ, we find:

$$\begin{aligned}\mu_{cg}^{(N)} &= \alpha_g \frac{\eta_z}{\beta_z} \ell_c = \rho_{cg}^{(N)} \ell_c, \\ \mu_{cg}^{(M)} &= \alpha_g \frac{\eta_z}{\gamma_z} \ell_c = \rho_{cg}^{(M)} \ell_c.\end{aligned} \tag{39}$$

We recapitulate the same functional form under a distinct series of assumptions. To obtain this solution, we used a convenient property by which a Bernoulli-subsampled negative binomial distribution remains negative binomial.

S1.3 The negative binomial model and its bursty extensions

Alternatively, the following model can produce a negative binomial stationary distribution of $\mathcal{X}$:

$$\varnothing \xrightarrow{k} B \times \mathcal{X} \xrightarrow{\gamma} \varnothing, \tag{40}$$

i.e., transcription events arrive at rate k, and each one induces a *burst* of activity, with the number of produced transcripts B drawn from a geometric distribution on $\mathbb{N}_0$ with mean b. In the parlance of Equation 2, the law of $\mathcal{X}$ is negative binomial with shape $\alpha = k/\gamma$ and mean $\mu = kb/\gamma$.

S1.3.1 "Cell size" as biological variation

To include dependence on z and the "cell size," while maintaining the assumption of constant shape parameter, we recall that the burst model arises in a particular limit of the two-state telegraph model [19]. The burst frequency k is the rate at which the gene turns on, whereas the burst size b is the average number of molecules produced while the gene is on. The burst size is proportional to the transcription initiation rate, which is in turn controlled by [RNAP]. By choosing the appropriate units and separating out the initiation term to yield $b_{cg} = b_{z,init}[\text{RNAP}]_c$, we obtain

$$\begin{aligned}\mu_{cg} &= \frac{k_g b_{cg}}{\gamma_g} = \alpha_g b_{z,init}[\text{RNAP}]_c \\ &= \frac{\alpha_g b_{z,init}}{C} C[\text{RNAP}]_c, \\ \rho_{cg} &:= \frac{\alpha_g b_{z,init}}{C}, \\ \ell_c &:= C[\text{RNAP}]_c.\end{aligned} \tag{41}$$

Next, we write down the two-species system:

$$\varnothing \xrightarrow{k} B \times \mathcal{N} \xrightarrow{\beta} \mathcal{M} \xrightarrow{\gamma} \varnothing, \tag{42}$$

which induces the a distribution with the following probability-generating function (PGF) [22]:

$$\begin{aligned}G(g_N, g_M; k, b, \beta, \gamma) &= \sum_{n,m=0}^{\infty} g_N^n g_M^m P(n,m) \\ &= \exp\left(k \int_0^{\infty} \left[\frac{1}{1 - bU(u_N, u_M, s; \beta, \gamma)} - 1\right] ds\right), \\ \text{s.t. } U(u_N, u_M, s; \beta, \gamma) &= \frac{\beta}{\beta - \gamma} u_M e^{-\gamma s} + \left(u_N - \frac{\beta}{\beta - \gamma} u_M\right) e^{-\beta s} \\ \text{and } u_i &:= g_i - 1.\end{aligned} \tag{43}$$

As in the generic case, $\mu_N = kb/\beta$ and $\mu_M = kb/\gamma$. The bivariate distribution no longer has a well-defined and unique α_g parameter; for consistency with the "shape of the negative binomial marginal" interpretation, we choose $\alpha := k/\beta$. Equation 43 typically cannot be evaluated in closed form, but may be approximated:

$$P(n, m; \alpha, \mu_N, \mu_M) = P_{\text{NB}}(n; \alpha, \mu_N) P(m|n; \alpha, \mu_N, \mu_M), \tag{44}$$

where $P(m|n)$ can be represented by a mixture of negative binomial distributions [23].

We maintain the assumptions of z and "cell size" dependence through b, and impose the assumption that k, β, and γ are gene-specific and constant. This yields:

$$\begin{aligned}\mu_{cg}^{(N)} &= \frac{k_g b_{z,init}}{\beta_g C} = \frac{\alpha_g b_{z,init}}{C} = \rho_{cg} \ell_c, \\ \mu_{cg}^{(M)} &= \frac{k_g b_{z,init}}{\gamma_g C} = \frac{\beta_g}{\gamma_g} \frac{\alpha_g b_{z,init}}{C} = \frac{\beta_g}{\gamma_g} \rho_{cg} \ell_c,\end{aligned} \tag{45}$$

where β_g/γ_g may be fit or estimated from the plug-in method of moments estimate $\overline{M}_g/\overline{N}_g$.

As before, if we suppose that k, γ, and β can differ between cell types, we are forced to define two distinct ρ parameters:

$$\begin{aligned}\mu_{cg}^{(N)} &= \rho_{cg}^{(N)} \ell_c \text{ s.t. } \rho_{cg}^{(N)} := \frac{k_z b_{z,init}}{C \beta_z}, \\ \mu_{cg}^{(M)} &= \rho_{cg}^{(M)} \ell_c \text{ s.t. } \rho_{cg}^{(M)} := \frac{k_z b_{z,init}}{C \gamma_z}.\end{aligned} \tag{46}$$

However, this highly generic form violates admissibility under the *scVI* model: some shape-like parameter should be constant. Maintaining $\alpha_g = k_g/\beta_g$, we yield the following formulation:

$$\begin{aligned} \mu_{cg}^{(N)} &= \rho_{cg}^{(N)} \ell_c \text{ s.t. } \rho_{cg}^{(N)} := \frac{\alpha_g b_{z,init}}{C}, \\ \mu_{cg}^{(M)} &= \rho_{cg}^{(M)} \ell_c \text{ s.t. } \rho_{cg}^{(M)} := \frac{\beta_g}{\gamma_z} \frac{\alpha_g b_{z,init}}{C}. \end{aligned} \tag{47}$$

In sum, if we assume each gene's splicing and degradation rates are constant across all cells, we use Equation 44 to evaluate likelihoods, with $\mu_N = k_g b_{cg}/\beta_g = \frac{\gamma_g}{\beta_g} \rho_{cg} \ell_c$, $\mu_M = \rho_{cg} \ell_c$, and a shared fit parameter $\alpha_g = k_g/\beta_g$. On the other hand, if we relax the assumption of constant γ_g, we use the same equation, with $\mu_N = \rho_{cg}^{(N)} \ell_c$ and $\mu_M = \rho_{cg}^{(M)} \ell_c$.

S1.3.2 "Cell size" as technical variation

Just as in Section S1.2.2, the univariate model stays negative binomial after Bernoulli technical noise:

$$\begin{aligned} \mu_{cg} &= \frac{k_g b_{cg}}{\gamma_g} p_c = \alpha_g b_z p_c, \\ &= \alpha_g \frac{b_z}{C} C p_c, \end{aligned} \tag{48}$$

i.e., the downsampling amounts to rescaling b. The *scVI* variables can be interpreted as follows:

$$\begin{aligned} \rho_{cg} &:= \alpha_g \frac{b_z}{C}, \\ \ell_c &:= C p_c. \end{aligned} \tag{49}$$

The two-species model is somewhat more complicated. Specifically, we find that the characteristic takes the following form:

$$\begin{aligned} bU(u_N, u_M, s; \beta, \gamma) &= b p_N u_N e^{-\gamma s} + b p_M u_M \frac{\beta}{\beta - \gamma} \left(e^{-\beta s} - e^{-\gamma s} \right) \\ &= b p_N \left[u_N e^{-\gamma s} + \frac{p_M}{p_N} u_M \frac{\beta}{\beta - \gamma} \left(e^{-\beta s} - e^{-\gamma s} \right) \right]. \end{aligned} \tag{50}$$

By evaluating u_N and u_M at zero, we can verify that the nascent and mature marginals separately follow the functional form in Equation 43 with burst sizes bp_N and bp_M. However, elsewhere, the integrand violates this form: for example, if we fix γ, there is no way to choose β so p_M/p_N will be canceled out at each s. This suggests the integral on $s \in [0, \infty)$ – i.e., the stationary PGF – does not match that of the original distribution.

As the current study primarily focuses on the recovery of biophysical, rather than technical parameters, we elide this problem by assuming $p_N = p_M$. This is a coarse approximation that presupposes the sequencing efficiencies of spliced and unspliced RNA are roughly equivalent. It is likely that the relevant parameters can be inferred, or approximated by a simple plug-in estimate [61].

Thus, under the assumption of $p_N = p_M = p$, we yield the law given in Equation [43] with the b variable set to bp_c. Enforcing a constant $\alpha_g = k_g/\beta_g$, we yield the following results:

$$\begin{aligned} \mu_{cg}^{(N)} &= \alpha_g \frac{b_z}{C} C p_c = \rho_{cg}^{(N)} \ell_c, \\ \mu_{cg}^{(M)} &= \alpha_g \frac{\beta_g}{\gamma_z} \frac{b_z}{C} C p_c = \rho_{cg}^{(M)} \ell_c, \end{aligned} \tag{51}$$

which recapitulates the results in the previous section.

S1.4 The zero-inflated negative binomial model and its multi-state interpretation

The default *scVI* generative model is zero-inflated negative binomial (ZINB), which has the following functional form:

$$\begin{aligned} P(x; \alpha, \mu, \pi) &= \pi P_{\mathrm{NB}}(x; \alpha, \mu) \text{ when } x > 0, \text{ and} \\ &= (1 - \pi) + \pi P_{\mathrm{NB}}(0; \alpha, \mu) \text{ when } x = 0. \end{aligned} \tag{52}$$

The quantity $\pi \in (0, 1)$ is the zero-inflation probability, which is computed using a neural network in the standard *scVI* pipeline.

Despite its popularity, the zero-inflated model is somewhat contentious [79], with a fair amount of evidence suggesting that zero-inflation is not necessary to summarize the distributions of molecule counts in single-cell RNA sequencing data [80]. Our previous mechanistic studies in relatively homogeneous cell types [61, 62] have not implicated zero-inflation as a significant effect.

More problematically, zero-inflation appears to be a purely *ad hoc* mathematical tool, and does not describe any specific biophysical process. We are aware of one report that attempts to motivate it [81]. However, we find this attempt to be unrealistic, as it conflates biological processes with experimental ones: the report describes a *biological* state that leads to *technical* non-identifiability, without motivating a chemical mechanism.

In principle, we *can* write down a system that yields a zero-inflated negative binomial stationary distribution, a simple three-state model [41]:

$$\begin{aligned} &\mathcal{G}_0 \underset{k_{10}}{\overset{k_{01}}{\rightleftarrows}} \mathcal{G}_1, \\ &\mathcal{G}_1 \underset{k_{21}}{\overset{k_{12}}{\rightleftarrows}} \mathcal{G}_2, \\ &\mathcal{G}_2 \xrightarrow{k_{init}} \mathcal{G}_2 + \mathcal{X}. \end{aligned} \tag{53}$$

We omit the downstream processes, which are generic and immaterial. Ostensibly, if $k_{01} = 0$, this reduces to the two-state model. If $k_{21}, k_{init} \to \infty$ such that k_{init}/k_{21} is finite, this further reduces to the bursty model with burst size $b = k_{init}/k_{21}$ and burst frequency $k = k_{12}$. In this limit, the instantaneous probability of being in state $\mathcal{G}_2$ vanishes.

If we impose $k_{10}, k_{01} \ll k_{12}$, but $k_{10}/k_{01} \sim \mathcal{O}(1)$, this system reduces to a two-component mixture, with $\pi = \frac{k_{10}}{k_{01}+k_{10}}$ giving the probability that the gene is in state $\mathcal{G}_1$. This recapitulates the functional form of the ZINB model. In sum, this probability law can emerge from a purely biological description of a system with two long-lived steady states; one of these steady states is inactive,

whereas the other exhibits bursts of activity. Analogous models have previously been proposed for co-regulation of gene loci [41], and can be leveraged to represent patterns of co-bursting [31]. For simplicity, we do not discuss this extension further, but the associated derivations would be largely analogous to those in Section S1.3.

S2 Simulations

S2.1 Generating parameters

To asses *biVI*'s ability to reconstruct transcriptional parameters when ground truth is known, data was simulated for three physical models of transcription: the bursty model, the constitutive model, and the model of extrinsic noise, derived in S1. For all models, parameters were generated for 2,000 genes across 20 cell types. First, baseline $\log_{10}$ model parameters were drawn from multivariate normal distributions for all 2,000 genes.

For the bursty model, mean burst size b, relative splicing rate β/k, and relative degradation rate γ/k were sampled from to the following trivariate normal distribution:

$$\begin{pmatrix} \log_{10} b \\ \log_{10} \frac{\beta}{k} \\ \log_{10} \frac{\gamma}{k} \end{pmatrix} \sim N\left[\begin{pmatrix} 0.2 \\ 0.5 \\ 0.4 \end{pmatrix}, \begin{pmatrix} 0.36 & 0.144 & 0.24 \\ 0.144 & 0.09 & 0.12 \\ 0.24 & 0.12 & 0.5 \end{pmatrix}\right].$$

For the constitutive model, parameters for relative splicing rates β/k and relative degradation rates γ/k were generated from the following bivariate normal distribution:

$$\begin{pmatrix} \log_{10} \frac{\beta}{k} \\ \log_{10} \frac{\gamma}{k} \end{pmatrix} \sim N\left[\begin{pmatrix} 0.497 \\ 0.711 \end{pmatrix}, \begin{pmatrix} 0.318 & 0.062 \\ 0.062 & 0.237 \end{pmatrix}\right].$$

Finally, for the extrinsic model, the negative binomial shape parameter α, relative splicing rate β/η, and relative degradation rate γ/η were generated from the following trivariate normal distribution:

$$\begin{pmatrix} \log_{10} \alpha \\ \log_{10} \frac{\beta}{\eta} \\ \log_{10} \frac{\gamma}{\eta} \end{pmatrix} \sim N\left[\begin{pmatrix} 2.53 \\ 0.31 \\ 0.25 \end{pmatrix}, \begin{pmatrix} 1.01 & 0.26 & -0.08 \\ 0.26 & 0.49 & 0.11 \\ -0.08 & 0.11 & 0.21 \end{pmatrix}\right].$$

The parameter distribution for the bursty and extrinsic models were derived from *Monod* fits to a cell type dataset (assuming no technical noise) [62]. The parameter distribution for the constitutive model was derived from a joint distribution of nascent and mature means.

After creating the baseline, we added random noise, distributed per $\epsilon \sim N(0, 0.05)$ to all $\log_{10} b$ and $\log_{10} \frac{\gamma}{k}$ for the bursty model, and $\log_{10} \frac{\beta}{k}$ and $\log_{10} \frac{\gamma}{k}$ for the constitutive and extrinsic models per cell type. Therefore, each cell type had slightly different parameters for non-marker genes. For consistency with default *scVI* learning assumptions, we did not change the relative splicing rate $\frac{\beta}{k}$ of the bursty model and the inverse dispersion parameter α of the extrinsic model: though different across genes, they were identical across all cell types.

Next, we imposed more realistic cell type structure by selecting marker genes. The number of marker genes per cell type was chosen for each cell type by drawing from a Poisson distribution with a mean of 45. Marker genes were non-overlapping. In the bursty model, marker gene burst sizes were augmented by $\Delta \sim N(1.0, 0.1)$ in the relevant cell type. In the constitutive model, marker gene parameters were modified by increasing the rate of transcription k, effected by subtracting $\Delta \sim N(1.0, 0.1)$ from each $\log_{10} \beta/k$ and $\log_{10} \gamma/k$. In the extrinsic model, marker gene parameters were modified by increasing the scale of transcription η, effected by subtracting $\Delta \sim N(1.0, 0.1)$ from each $\log_{10} \beta/\eta$ and $\log_{10} \gamma/\eta$.

S2.2 Sampling count data

10,000 cells spread across the 20 cell types were simulated for each model. The number of cells per cell type was assigned using a Dirichlet-multinomial distribution: a probability vector $\mathbf{p}$ was drawn from a Dirichlet distribution parameterized by a vector of 20 equal elements, then cell types for each of the 10,000 cells were assigned using a multinomial distribution with probabilities $\mathbf{p}$. Nascent and mature counts for each of the 10,000 cells were then sampled from model distributions (Equation 13 for bursty data, Equation 7 for constitutive data, and Equation 11 for extrinsic data) using parameters specific to the cell's assigned cell type.

S3 Network architecture

The *biVI* architecture used for all figures and analyses in this study employs two encoders of 3 fully connected, 128 node layers to map from input space $\mathbb{R}^{2N_G}$, where N_G is the number of genes, to latent means $z_\mu \in \mathbb{R}^{10}$ and latent standard deviations $z_\sigma \in \mathbb{R}^{10}$. The decoder, comprising 3 fully connected layers of 128 nodes, maps from sampled latent space $z_n \in \mathbb{R}^{10}$ back to the original input space $\mathbb{R}^{2N_G}$. There are ReLU activation functions between each layer of the encoder networks and decoder network. Furthermore, a softmax is applied to the output layer of the decoder to produce normalized expression levels $\rho_{cg}^{(N)}$ and $\rho_{cg}^{(M)}$ for nascent and mature expression levels, respectively. $\rho_{cg}^{(N)}$ and $\rho_{cg}^{(M)}$ are multiplied by observed sequencing depth before conditional likelihoods (reconstruction errors) are calculated. For the bursty and extrinsic models, the architecture includes parameters $\alpha \in \mathbb{R}^{N_G}$ that are updated during inference. For the constitutive model, only nascent and mature means parameterize the conditional likelihood, so α is unused.

S4 Benchmarking metrics

The following clustering metrics were calculated for simulated data on the latent spaces produced by each model. Ground truth simulated cell type was used as cluster assignment. Let N_C be the total number of cells and J be the number of clusters. Let $z_c \in \mathbb{R}^d$ be the low-dimensional representation of cell c, n_κ be the number of cells in each cluster κ (such that $\sum_\kappa n_\kappa = N_C$), and $\mu_\kappa \in \mathbb{R}^d$ be the mean of low-dimensional representations of cells in cluster κ.

Average intra-cluster distance (average ICD) Average intra-cluster distance was found by calculating the Euclidean distance between each cell's low-dimensional representation and its cluster mean's low-dimensional representation. First, the average intra-cluster distance for each cluster κ was found:

$$\mathrm{ICD}_{c_\kappa} = \|z_{c_\kappa} - \mu_\kappa\|_2, \tag{54}$$

$$\overline{\mathrm{ICD}_\kappa} = \frac{1}{n_\kappa} \sum_{c_\kappa=1}^{n_\kappa} \mathrm{ICD}_{c_\kappa} \quad \forall\ c_\kappa \in \kappa. \tag{55}$$

Then, the average over all clusters was computed:

$$\text{average ICD} = \frac{1}{J} \sum_{\kappa=1}^{J} \overline{\mathrm{ICD}_\kappa}. \tag{56}$$

For clustering on the latent space, ideally the underlying cell types should be fairly "condensed", with a low average ICD.

Average intra-cluster variance (average ICV): The intra-cluster variance was calculated for each cluster κ as the variance in intra-cluster distances (ICDs) for all cells in cluster κ:

$$\mathrm{ICV}_\kappa = \frac{1}{n_\kappa} \sum_{c_\kappa=1}^{n_\kappa} (\mathrm{ICD}_{c_\kappa} - \overline{\mathrm{ICD}_\kappa})^2 \quad \forall\ c_\kappa \in \kappa. \tag{57}$$

Then, the average over all clusters was computed:

$$\text{average ICV} = \frac{1}{J} \sum_{\kappa=1}^{J} \mathrm{ICV}_\kappa. \tag{58}$$

For clustering on the latent space, ideally the average ICV should be low: having a high variation in spread tends to violate the assumptions of typical algorithms.

Average inter-cluster distance (average INCD): The distance between each cluster mean and all other cluster means was found. The average was computed over all these distances:

$$\text{average INCD} = \frac{1}{J^2 - J} \sum_{y=1}^{J} \sum_{\substack{x=1 \\ x \neq y}}^{J} \|\mu_x - \mu_y\|_2. \tag{59}$$

For clustering on the latent space, ideally the average INCD should be relatively high, to facilitate discrimination between populations.

Scaled inter-cluster distance (INCD): The scaled inter-cluster distance was found by dividing the average inter-cluster distance by the average intra-cluster distance:

$$\text{scaled INCD} = \frac{\text{average INCD}}{\text{average ICD}}. \tag{60}$$

As with the average INCD, ideally this quantity to be relatively high.

Silhouette score : The silhouette score was calculated as follows:

$$\frac{b-a}{\max(a,b)},$$

where a is the average intra-cluster distance and b is the average nearest-cluster distance, or mean of the distance between each sample and the nearest cluster that it is not a part of. Silhouette score was implemented using the function `sklearn.metrics.silhouette_score` [82]. Ideally the silhouette score should be high, to accentuate the differences between clusters.

Nearest neighbor percentage : Nearest neighbor percentages were calculated for each cell by dividing the number of the n_κ nearest cells (in low-dimensional space) in the same cluster κ as the cell by the total number of cells, n_κ, in that cluster.

The computation was implemented using `sklearn.neighbors.KNeighborsClassifier` [82]. Ideally the nearest neighbor percentage should be high, as it helps interpret neighborhood relationships in the latent space as cell type similarity.

Mean squared error : The mean squared error (MSE) between reconstructed parameters and ground truth parameters over all genes was calculated. Ideally this quantity should be low, as it confirms that the autoencoder is not merely learning distribution shapes (potentially overfitting or underfitting due to distributional degeneracies), but obtaining quantitative information about the parameter values.

S5 Supplementary Figures

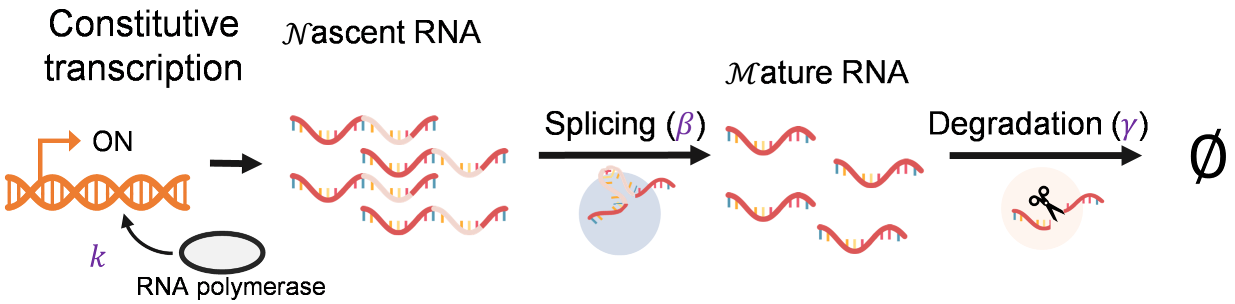

Figure S1: Diagram of constitutive transcription. Transcription occurs at constant rate k to produce nascent RNA molecules. Next, splicing occurs according to a Poisson process with rate β to produce mature RNA molecules, which are degraded according to a Poisson process with rate γ.

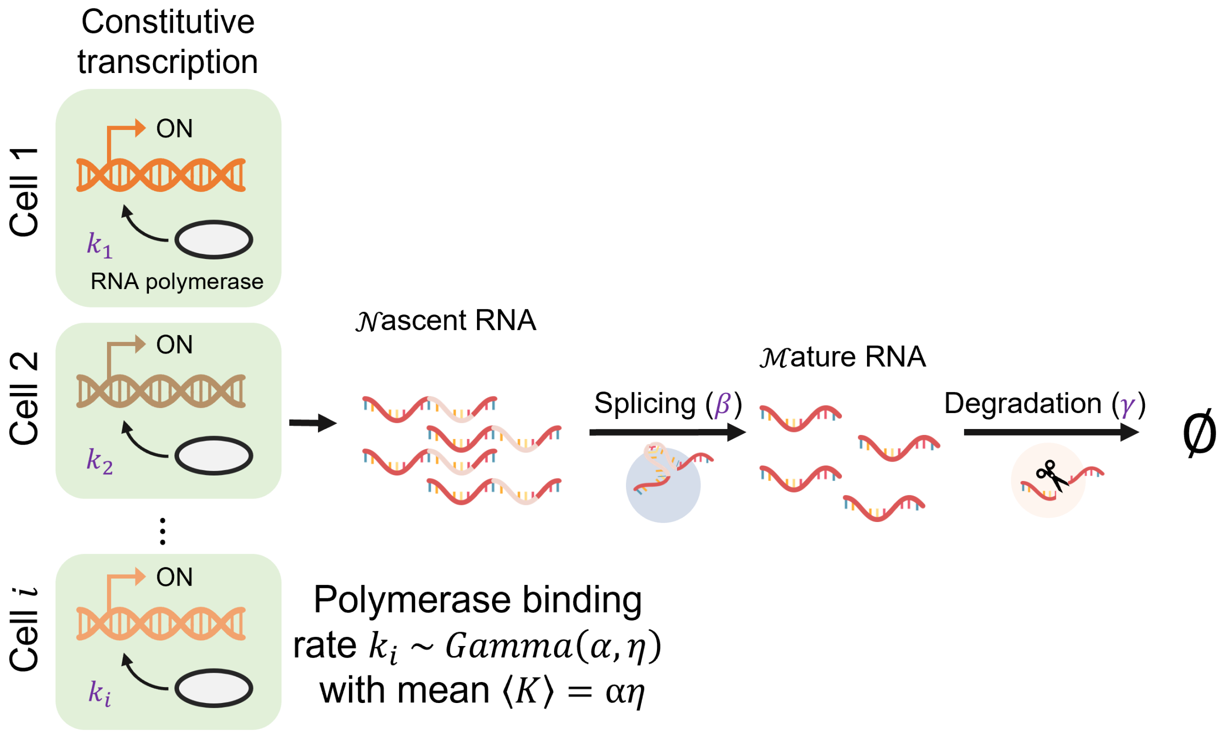

Figure S2: Diagram of the extrinsic model of transcription. Transcription across cells of the same cell type occurs according to a Poisson process with cell-specific rates $k \sim Gamma(\alpha, \eta)$ to produce nascent RNA molecules. Next, splicing occurs according to a Poisson process with rate β to produce mature RNA molecules, which are degraded according to a Poisson process with rate γ.

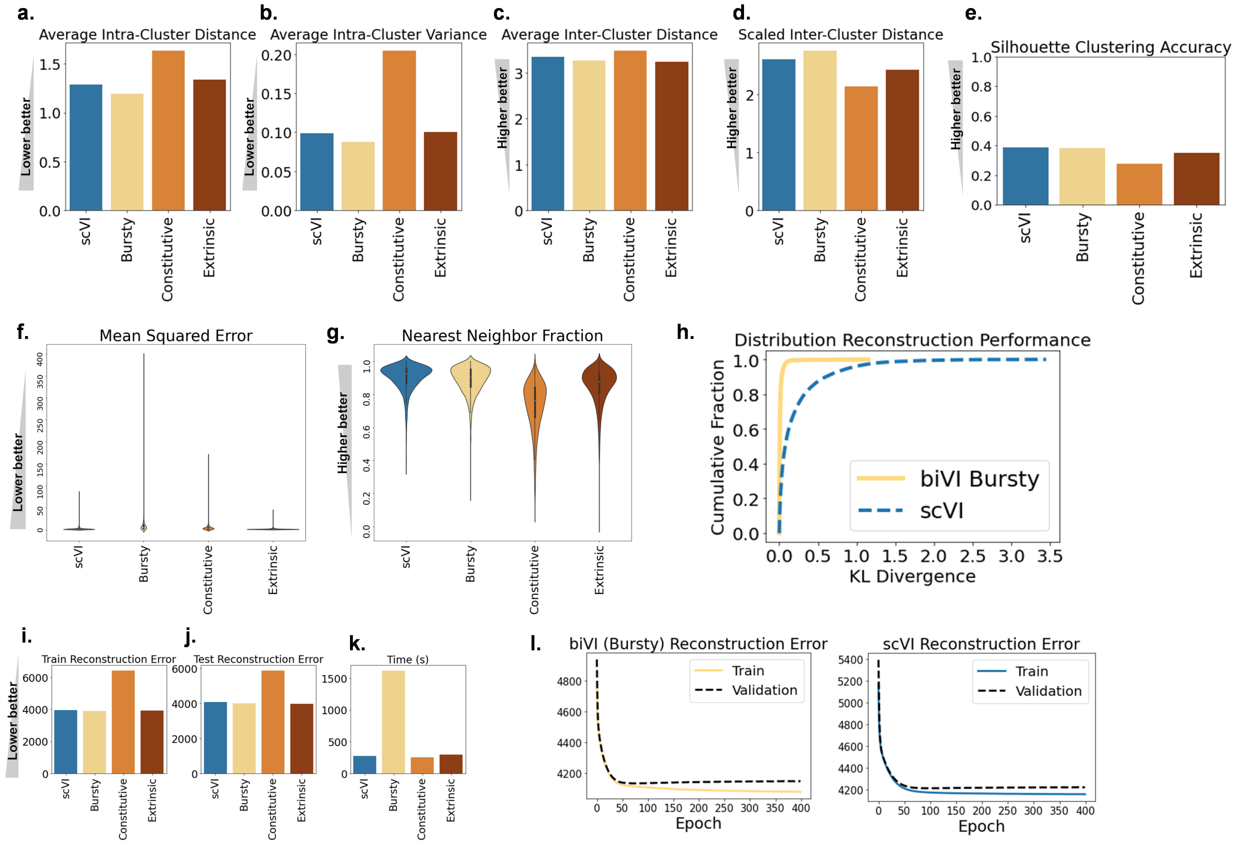

Figure S3: Clustering metrics and testing performance performance on bursty simulated data, with simulated ground truth cell type used as cluster label. Models trained on 7,200 cells with 800 validation cells for 400 epochs. All metrics were calculated on 2,000 held-out testing cells. **a.** Intra-cluster distance (average over all clusters). **b.** Intra-cluster variance (average over all clusters). **c.** Inter-cluster distance (average over all clusters). **d.** Average inter-cluster distance scaled by average intra-cluster distance. **e.** Average silhouette score (over all cells). **f.** Mean squared error between reconstructed nascent and mature means and observed nascent and mature counts (average over all cells). **g.** Fraction of nearest neighbors (closest cells in latent space) in the same cluster as each cell (average over all cells). **h.** Empirical cumulative distribution function (eCDF) of KL divergence between reconstructed distributions (using *biVI* with bursty likelihood) and simulated ground truth distributions for all 2,000 testing cells, evaluated in individual cell types. **i.** Reconstruction error (negative log likelihood under each model) after 400 training epochs on training data. **j.** Reconstruction error after 400 training epochs on testing data. **k.** Time in seconds to train each model for 400 epochs. **l.** Reconstruction error (negative log likelihood under each model) on train and validation data over training epochs for *biVI* with the bursty likelihood and *scVI* with independent negative binomial likelihoods.

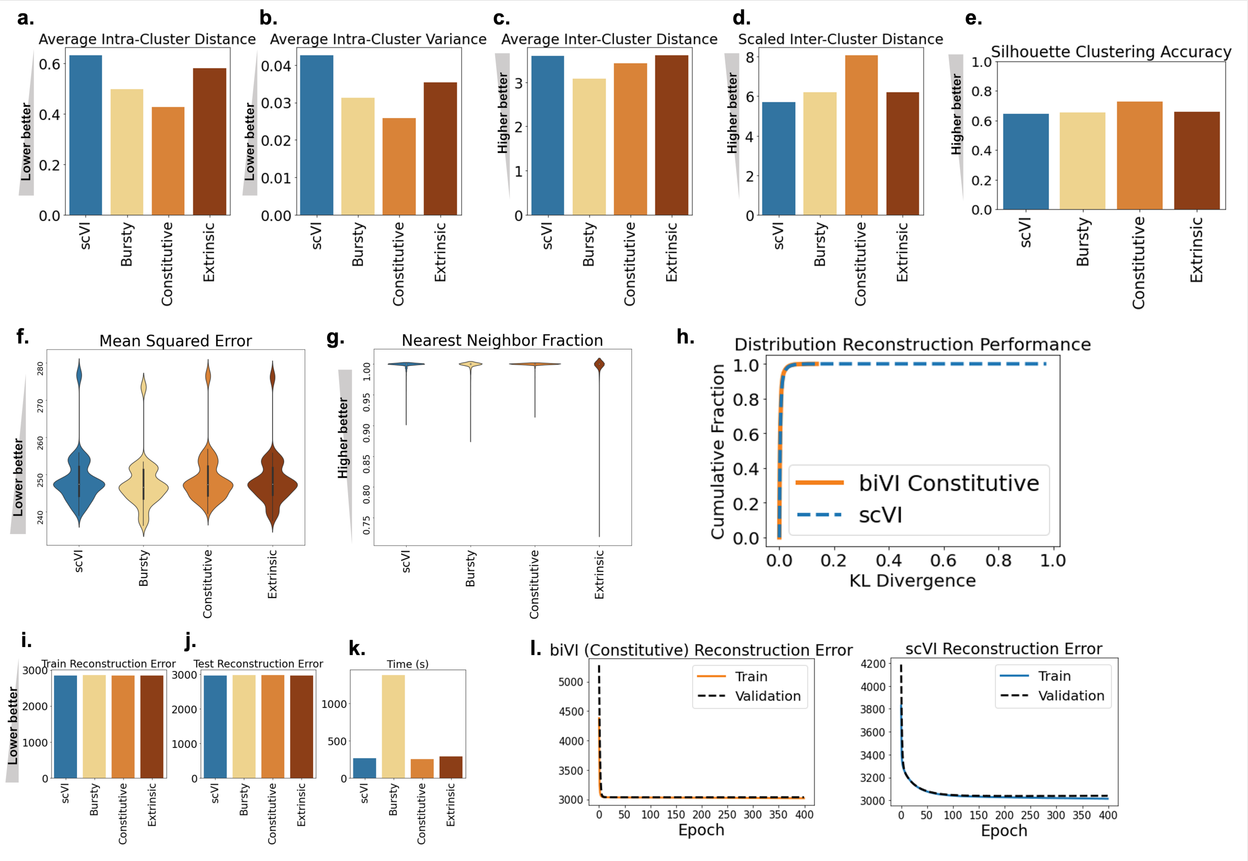

Figure S4: Clustering metrics and testing performance performance on constitutive simulated data, with simulated ground truth cell type used as cluster label. Models trained on 7,200 cells with 800 validation cells for 400 epochs. All metrics were calculated on 2,000 held-out testing cells. All conventions as in Fig. S3.

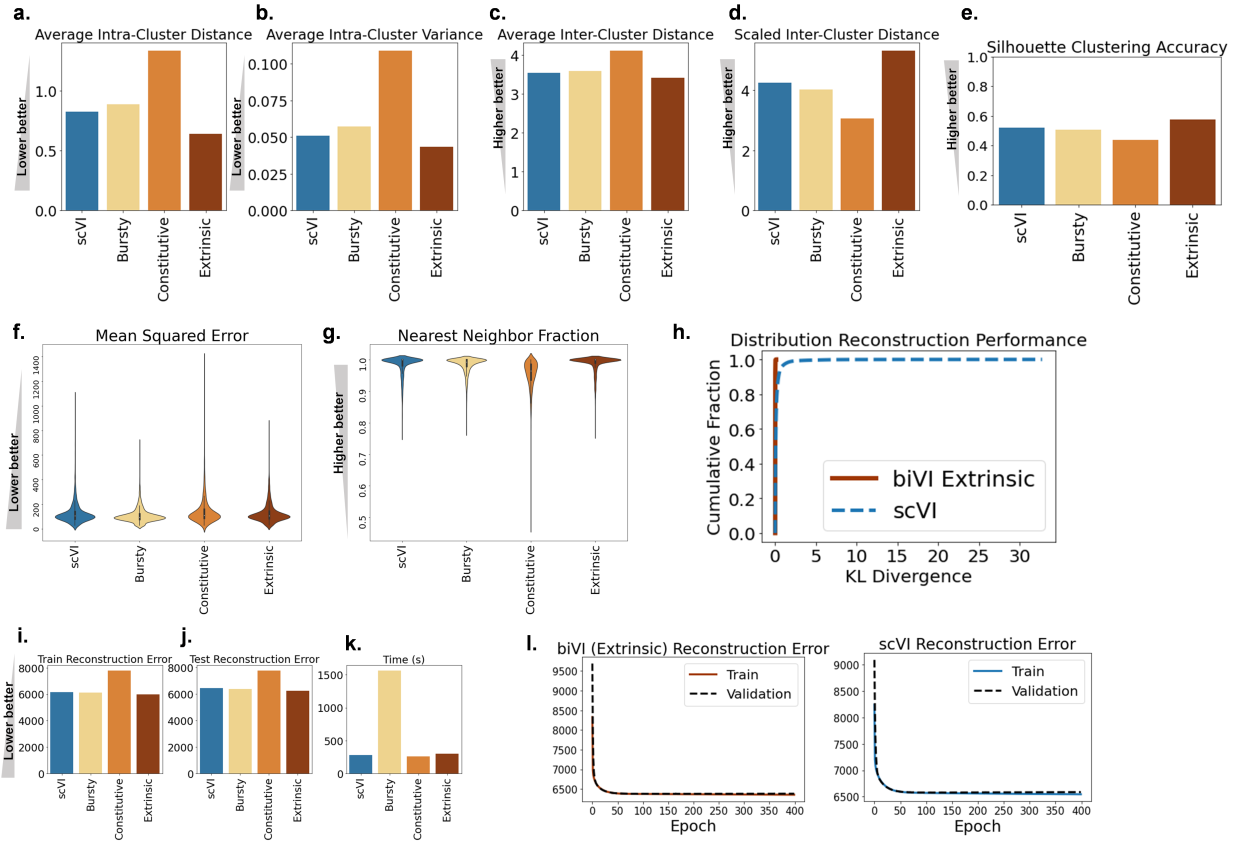

Figure S5: Clustering metrics and testing performance performance on extrinsic simulated data, with simulated ground truth cell type used as cluster label. Models trained on 7,200 cells with 800 validation cells for 400 epochs. All metrics were calculated on 2,000 held-out testing cells. All conventions as in Fig. S3.

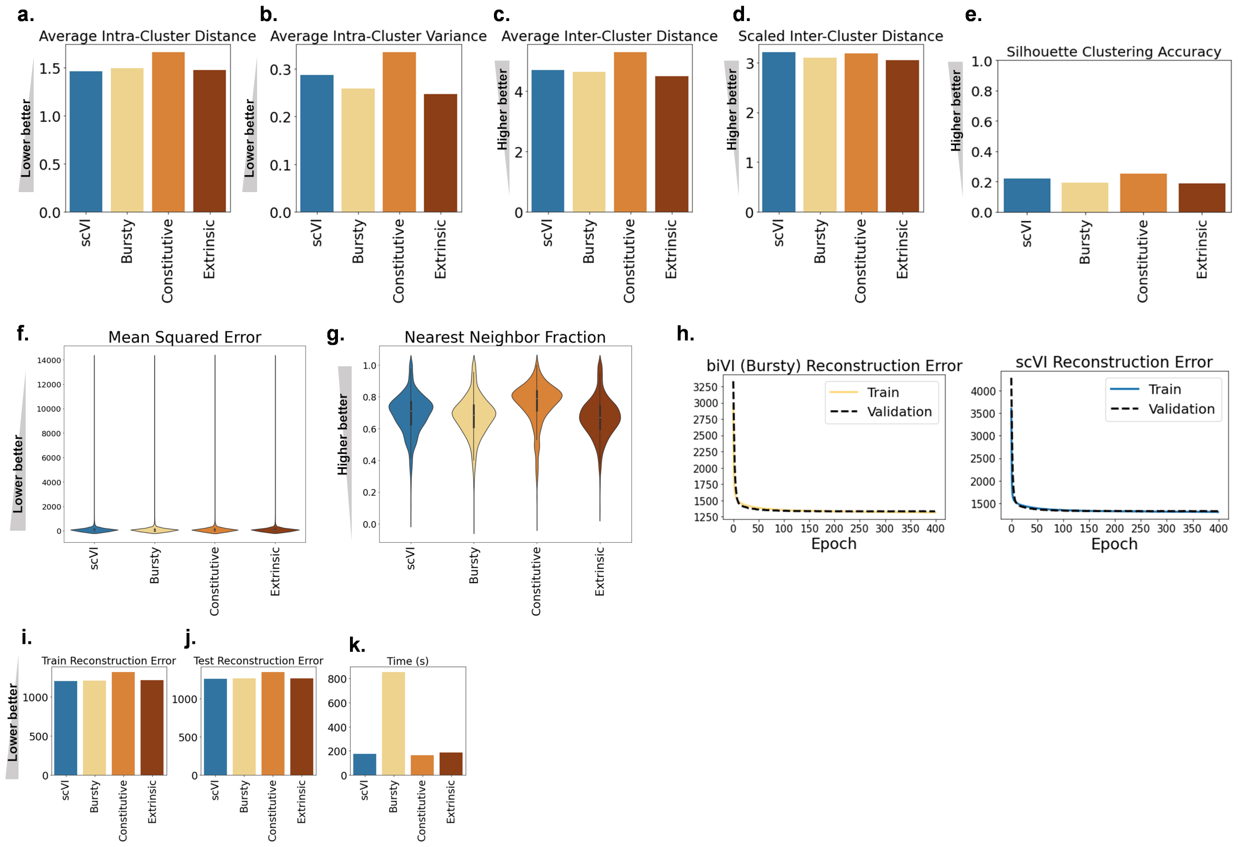

Figure S6: Clustering metrics and testing performance performance on Allen data sample B08, with annotated subclass used as cluster labels. Models trained on 4,622 cells with 513 validation cells for 400 epochs and all metrics calculated on 1,283 held-out testing cells. **a.** Intra-cluster distance (average over all clusters). **b.** Intra-cluster variance (average over all clusters). **c.** Inter-cluster distance (average over all clusters). **d.** Average inter-cluster distance scaled by average intra-cluster distance. **e.** Average silhouette score (over all cells). **f.** Mean squared error between reconstructed nascent and mature means and observed nascent and mature counts (average over all cells). **g.** Fraction of nearest neighbors (closest cells in latent space) in the same cluster as each cell (average over all cells). **h.** Reconstruction error (negative log likelihood under each model) on train and validation data over training epochs for *biVI* with the bursty likelihood distribution and *scVI* with independent negative binomial likelihood distributions. **i.** Reconstruction error (negative log likelihood under each model) after 400 training epochs on training data. **j.** Reconstruction error after 400 training epochs on testing data. **k.** Time in seconds to train each model for 400 epochs.
